# Contribution of Specific Residues of the β-Solenoid Fold to HET-s Prion Function, Amyloid Structure and Stability

**DOI:** 10.1371/journal.ppat.1004158

**Published:** 2014-06-12

**Authors:** Asen Daskalov, Matthias Gantner, Marielle Aulikki Wälti, Thierry Schmidlin, Celestine N. Chi, Christian Wasmer, Anne Schütz, Johanna Ceschin, Corinne Clavé, Sandra Cescau, Beat Meier, Roland Riek, Sven J. Saupe

**Affiliations:** 1 Institut de Biochimie et de Génétique Cellulaire, Unité Mixte de Recherche 5095, Centre National de la Recherche Scientifique Université de Bordeaux, Bordeaux, France; 2 Laboratory of Physical Chemistry, Eidgenössische Technische Hochschule (ETH) Zürich, Zürich, Switzerland; University of Alberta, Canada

## Abstract

The [Het-s] prion of the fungus *Podospora anserina* represents a good model system for studying the structure-function relationship in amyloid proteins because a high resolution solid-state NMR structure of the amyloid prion form of the HET-s prion forming domain (PFD) is available. The HET-s PFD adopts a specific β-solenoid fold with two rungs of β-strands delimiting a triangular hydrophobic core. A C-terminal loop folds back onto the rigid core region and forms a more dynamic semi-hydrophobic pocket extending the hydrophobic core. Herein, an alanine scanning mutagenesis of the HET-s PFD was conducted. Different structural elements identified in the prion fold such as the triangular hydrophobic core, the salt bridges, the asparagines ladders and the C-terminal loop were altered and the effect of these mutations on prion function, fibril structure and stability was assayed. Prion activity and structure were found to be very robust; only a few key mutations were able to corrupt structure and function. While some mutations strongly destabilize the fold, many substitutions in fact increase stability of the fold. This increase in structural stability did not influence prion formation propensity *in vivo*. However, if an Ala replacement did alter the structure of the core or did influence the shape of the denaturation curve, the corresponding variant showed a decreased prion efficacy. It is also the finding that in addition to the structural elements of the rigid core region, the aromatic residues in the C-terminal semi-hydrophobic pocket are critical for prion propagation. Mutations in the latter region either positively or negatively affected prion formation. We thus identify a region that modulates prion formation although it is not part of the rigid cross-β core, an observation that might be relevant to other amyloid models.

## Author Summary

Prions are infectious protein particles causing fatal diseases in mammals. Prions correspond to self-perpetuating amyloid protein polymers. Prions also exist in fungi where they behave as cytoplasmic infectious elements. The [Het-s] prion of the fungus *Podospora anserina* constitutes a favorable model for the analysis of the structural basis of prion propagation because a high resolution structure of the prion form of [Het-s] is available, a situation so far unique to this prion model. We have analyzed the relation between [Het-s] structure and function using alanine scanning mutagenesis. We have generated 32 single amino acid variants of the prion forming domain and analyzed their prion function *in vivo* and structure by solid-state NMR. We find that the PFD structure is very robust and that only a few key mutations affect prion structure and function. In addition, we find that a C-terminal semi-flexible loop plays a critical role in prion propagation although it is not part of rigid amyloid core. This study offers insights on the structural basis of prion propagation and illustrates that accessory regions outside of the amyloid core can critically participate in prion function, an observation that could be relevant to other amyloid models.

## Introduction

Amyloids are protein aggregates with a cross-β structure. Amyloid folds are gradually being recognized as important components in the structural landscape of peptides and proteins. The amyloid fold has been envisioned as a generic primordial fold from which globular folds had to emancipate in prebiotic times to attain structural and functional diversification into globular proteins [1], [2], [3], [4], [5]. Amyloid folds also fulfill a variety of biological functions in relation to their specific structural properties [2], [6], [7]. Importantly, amyloids represent the underlying cause of a number of age-related protein deposition diseases which impose a major burden to modern societies [8], [9]. Yet, the determinants that govern amyloid folding and stability are much less well understood than in the case of globular proteins in part because of the scarcity of available high resolution structures of amyloid proteins.

Amyloids have the inherent property of being self-perpetuating and as such can represent the mechanistic basis for prion formation [10], [11], [12], [13]. Many prions are amyloids that are self-perpetuating *in vivo*. Amyloid prions cause fatal neurodegenerative diseases in mammals and can be encountered as epigenetic elements in fungi [14], [15]. The [Het-s] prion of the filamentous fungus *Podospora anserina* represents an example of such fungal prions [16], [17], [18]. Highly prevalent in nature, [Het-s] is involved in a non-self recognition process that takes place when cells of unlike genotype undergo fusion [16], [19]. This process termed heterokaryon incompatibility leads to the cell death of the mixed fusion cells. The *het-s* gene exists as two incompatible alleles termed *het-s* and *het-S*. When a *het-s* and a *het-S* strain are confronted, the incompatibility cell death response leads to the formation of a macroscopic demarcation line termed barrage. It is proposed that this cell death reaction might have a more general function in fungal defense [20], [21], [22]. Strains of the *het-s* genotype exist as two alternate epigenetic states: [Het-s*] (the non-prion-state) and [Het-s] (the prion state). Transition to the prion state can occur spontaneously at a low rate or can be induced systematically by contact with a prion-infected ([Het-s]) strain. The prion form then invades the fungal hyphal network at a rate an order of magnitude higher than the linear growth rate of the fungus [23]. Incompatibility is only triggered when HET-s is in the prion form ([Het-s]). Thus in this system, the prion state corresponds to the active state of the protein. The [Het-s] prion also displays a specific effect in the sexual cycle, where presence of the prion form leads to specific abortion of the *het-S* spores in a sexual cross, a process designated spore-killing [24].

The HET-s protein displays two distinct domains, an N-terminal globular domain termed HeLo and a C-terminal prion forming domain [25], [26]. The PFD encompassing residues 218 to 289 is natively unfolded in the soluble form of the proteins and adopts a specific β-solenoid fold in the amyloid prion form of the protein [25], [27], [28], [29]. Cell death is triggered when [Het-s] interacts with HET-S because the HET-s PFD templates the folding of the homologous region of HET-S into the β-solenoid fold which in turn induces a refolding of the globular domain and exposition of a N-terminal hydrophobic helix which targets the cell membrane [30], [31], [32]. A second mode of activation of HET-S, apparently involves the NWD2 STAND protein encoded by the gene adjacent to HET-S which contains an N-terminal region homologous to the PFD region [21], [33].

**Figure 1:**
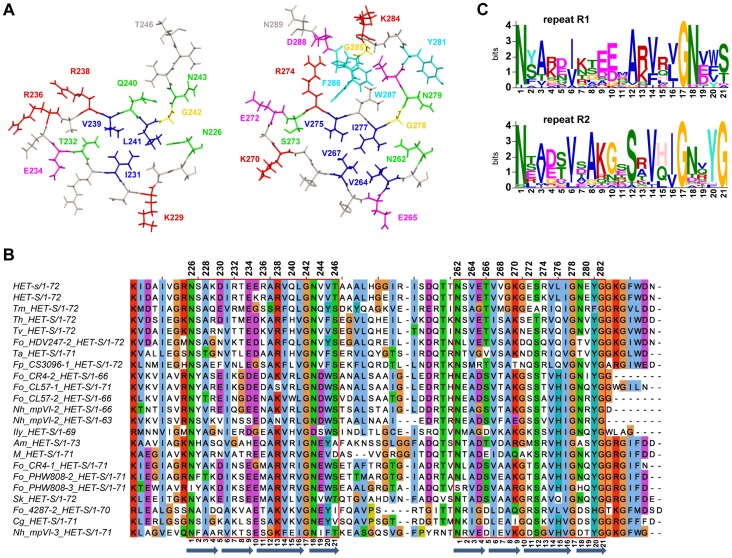
Structure and sequence conservation of the HET-s prion forming domain. **A.** The structure of the first pseudo repeat (residue 226–246) as well as the second repeat and the C-terminal loop region (residue 262–289) are given (after PDB 2KJ3). The 32 residues that have been mutated are coloured. Colour coding is as follows, polar residues in green, hydrophobic in blue, aromatic in cyan, positively charge in red, negatively charged in magenta and glycine in yellow. **B.** Sequence alignment of the PFD region of various HET-S homologs from various fungal species. The 21 amino acid repeats are boxed. The position of the β-strands of the β-solenoid core is represented as blue arrows **C.** Consensus sequence of the first and second repeats of the HET-S homologs presented in the alignment in B. The consensus was generated using MEME. In this graphical representation, the size of the letter reflects the level of conservation of the corresponding residue, the scale is given in information content measured in bits.

A high-resolution solid state NMR structure of the amyloid form of the HET-s PFD (HET-s(218–289) based on more than 2500 distance constraints has been reported [28], [29]. This domain adopts a β-solenoid fold in which β-strands delimit a triangular hydrophobic core. The domain is composed of two 21 amino acid long pseudo-repeats each of which forms one layer of β-strand in the β-solenoid structure, the two repeats are connected by a 15 amino acid long flexible loop. At the C-terminus of the domain, a C-terminal loop folds back onto the core region and forms a semi-hydrophobic pocket which can be considered as an extension of the hydrophobic core [28], (Figure 1A). The β-solenoid fold contains two aspargine ladders at the beginning of the first and last β-strand of each repeat and three salt bridges per monomer. The hydrophobic core contains essentially aliphatic residues and serine and threonine residues residing in different β-strand layers and forming a hydrogen bond within the core. A 8.5 Å cryo-EM model of HET-s(218–289) fibrils has also been reported and largely agrees with the ssNMR data. HET-s(218–289) are singlet fibrils with a left-handed twist and a helical pitch of 410 Å [34].

Sequence comparisons suggest an evolutionary conservation of the β-solenoid fold [35], [36]. Characterization of a HET-S homolog of a different fungal species, the plant pathogen *Fusarium graminearum* revealed a conservation of the β-solenoid fold and of the prion formation ability [37], [38]. Thus, the HET-s PFD sequence has been evolutionarily shaped to adopt this fold and the overall fold and the prion forming ability have been conserved over an extended evolutionary period. Perhaps, as a consequence of this evolutionary process, the HET-s PFD sequence does not to lead to the formation of amyloid polymorphs in physiological conditions as generally observed for disease causing amyloids or for yeast prions [13], [39]. HET-s(218–289) adopts the same structure *in vitro* and when forming inclusion bodies during heterologous expression in *E. coli* [40]. Only at highly acidic pH, when the native β-solenoid fold cannot be attained, HET-s(218–289) adopts an alternate non-infectious amyloid structure [34], [41], [42], [43], [44]. Alanine scanning approaches have been widely used to analyze folding and stability of globular proteins and such approaches have also been used in the context of amyloid fibril formation and stability [45], [46]. In the case of yeast prion proteins such as the well studied Sup35 model, the applicability of such approaches is complicated by the absence of a full structure model, the primary sequence independence in prion formation and the existence of multiple prion strains corresponding to different amyloid polymorphs [47], [48], [49]. Nevertheless, a recent study has revealed the importance of a glycine pair in [*PSI* ^+^] prion formation as a structural determinant in the soluble form of the PFD [50]. The HET-s model constitutes a favorable system for such studies because of the lack of prion strain variants and the availability of a high resolution structure. A previous study has revealed the functional importance of the β-strand elements in the β-solenoid structure using a proline mutant approach [27]. Here, we relied on the more subtle alanine scanning approach to analyze the functional and structural role of specific residues and structural components of the HET-s PFD fold.

## Results

### Evolutionary conservation of the PFD region in various fungal species

Previous studies have analyzed the evolutionary conservation of the β-solenoid fold [21], [35], [36], [37], [38]. Due to the rapid increase in the number of fully sequenced fungal genomes, many additional HET-S homologs sequences have become recently available, we have thus conducted a database search for HET-s/HET-S homologs in current fungal genome sequences. We could identify a total of 51 het-s/*het-S* homologs (file S1). Based on residues found at 33 of the Helo domain which is known to define HET-s and HET-S allele specificities [51], [52], the homologs found in the other species are of the HET-S, rather than HET-s-type (Figure S1), as suggested previously [18], [21], [30], [37]. Some species like *Nectria haematococca* and some strains of the *Fusarium oxysporum* species complex contained up to 4 *het-S* paralogs. The majority of the sequences showed conservation of the 21 amino acid repeat regions (R1 and R2) but in a fraction of the sequences there was a two amino acid deletion in the first repeated motif (R1). Figure 1B shows an alignment of the 22 non redundant sequences for which both 21-amino acid repeats are conserved. The level of identity between the sequences is in the range of 30% which is about the level of identity between the *P. anserina* HET-s(218–289) and *F. graminearum* FgHET-s(218–289) sequences that were previously found to display closely related structures [38]. It can thus be reasonably inferred that the sequences presented in the alignment share similar folds, as previously suggested by homology modeling approaches [35], [36]. The positions showing strict conservation are two of glycines (G242/G278, position 17 of the 21 aa repeat) located in the arc between the third and fourth β-strand of each rung, (Figure 1A and B). The N226/N262 asparagine pair (forming the asparagine ladder at the start of the first β-strand, position 1) was also conserved although some exceptions occurred. The second asparagine ladder (N243/N279, position 18) is not as strongly conserved as the first ladder. There is also a strong conservation of the hydrophobic residues in the core, large hydrophobic residues are found at core positions 231/267 (position 6), 239/275 (position 14) and 241/277 (position 16). There is an interesting trend of mutual exclusion of large hydrophobic side chains at position 228 and 264 (position 3), suggesting that the presence of large residues at both positions might lead to sterical hindrance. Interestingly, two sequences (Fo_4287-2 and Cg_HET-S) constitute exceptions in that regard with an isoleucine found both at 228 and 264. But in these sequences, an Ala residue is found in 231 which might allow for accommodation of the larger residues in position 3 (*i.e.* residue 231 in Figure 1A). Although the residues forming the three salt bridges identified in HET-s (in positions 4, 9 and 11) are not strictly conserved, there is an overall preference for favorable charge interactions at these positions. In the 22 listed sequences, favorable charge combination occurs in 13 and 15 sequences at position 4 and 9 respectively while repulsive interactions are never found. At position 11, favorable charge interactions are less conserved and occur in five sequences. Also of note, is the conservation of the glycine-rich C-terminal loop region encompassing the W287 and F286 aromatic residues. Conservation occurs not only on the aromatic positions but also the glycines and the charged residues (K284 and D288) are strongly conserved. This region is however entirely missing in a fraction of the sequences. Of note is the fact that the sequences missing this region are found in species that contain other HET-S paralogs that do display the loop region. The conservation is poor in the central loop region between R1 and R2. This region generally contains glycine residues and the length of the loop region varies from 12 to 18 residues. It was shown experimentally that three to five amino acid deletions in the loop region could be tolerated without affecting [Het-s] and HET-S function [26], [27].

In order to achieve an overall comparison of the two repeats, we devised a separate consensus sequence for repeats R1 and R2 using MEME [53] (Figure 1C). The consensus sequence reveals some marked differences. In the turn at position 10, there is a preference for a Glu in R1 and a Gly residue in R2. In position 12, there is a preference for an Ala and Ser in R1 and R2 respectively, which is reflected by the reciprocal distribution in position 8. Also, there is a strong conservation of the Gly at the end of R2 in the region leading into the C-terminal semi-hydrophobic pocket. The charge complementary mentioned above is also apparent in these consensus sequences; with a preference in R1 for positive charges in position 4 and negative charges in 9 and the opposite preference in R2. This differential conservation of specific residues in the first and the second repeat suggest that both repeats are not strictly functionally equivalent.

Globally, many aspects of the sequence conservation and variation in HET-s homologs are well explained by the structural information available. Yet, the role of certain features such as for instance the conservation of the Arg residue at position 13 is not directly apparent.

### Effect of alanine scanning mutagenesis on [Het-s] activity *in vivo*

**Figure 2:**
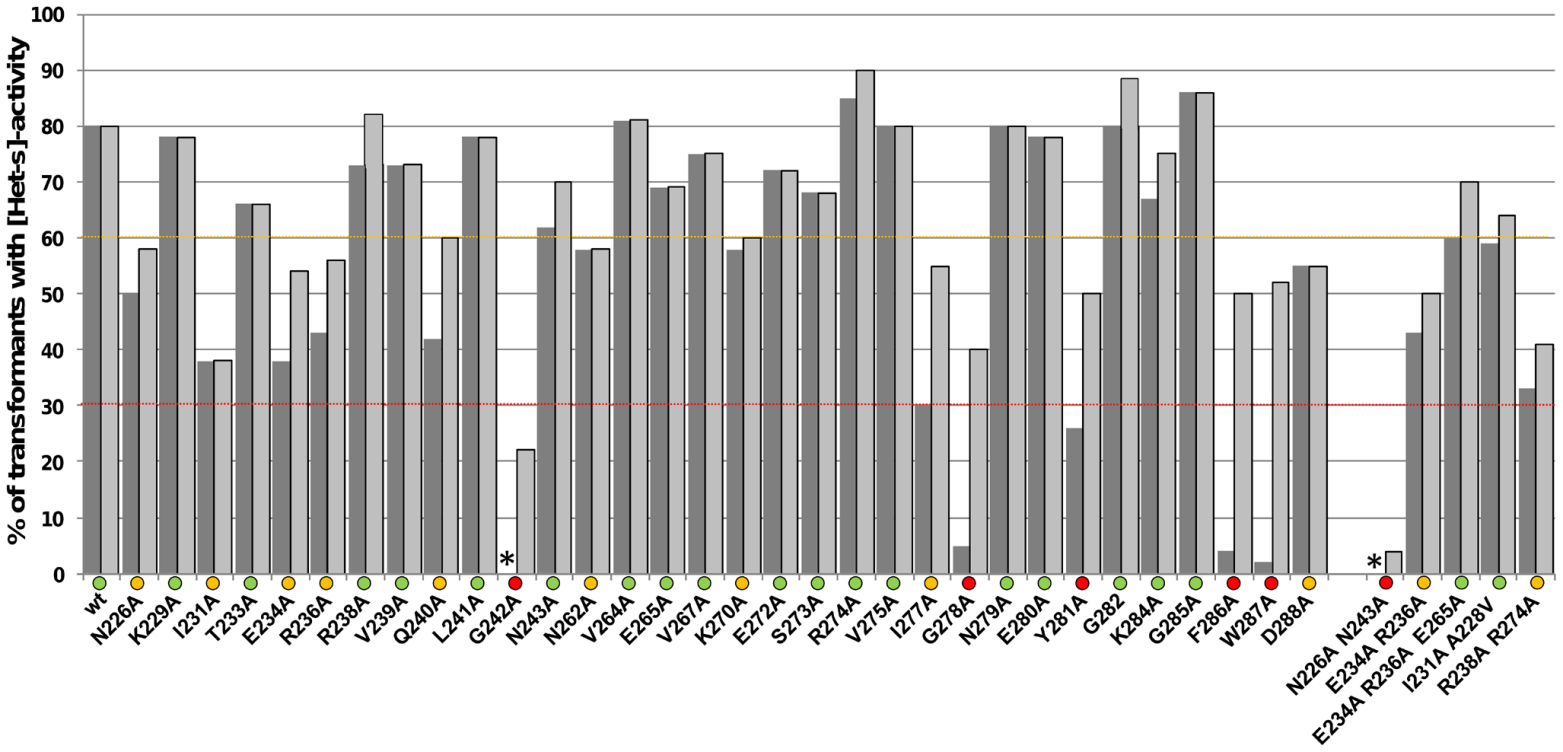
Effect of alanine mutations on [Het-s] activity *in vivo*. *Δhet-s* strain was transformed with the given mutants and the fraction of transformants producing a barrage reaction to [Het-S] (dark grey) and able to convert a [Het-s*] to the prion phenotype (light grey) is given in %. The mutants were grouped into three functional groups: mutants with wild-type or close to wild-type activity (>60%) were labeled by a green dot, slightly affected mutants (>30, <60%) were labeled by an orange dot, and strongly affected mutants (<30%) by a red dot, respectively. The “*” sign indicates that no transformant displayed the given activity.

We set out to analyze the structure-function relation in the HET-s PFD by generating a series of 32 alanine mutants along the HET-s PFD and by testing their prion activity *in vivo* in *Podospora anserina*. Based on the sequence conservation described above the β-strand region and the C-terminal pocket were specifically targeted (Figure 1A). The mutation coverage amounts to one mutation every 1.8 residue in these regions. Mutations were introduced in a plasmid expressing full length HET-s and the corresponding plasmid was introduced into a *Δhet-s* strain as previously reported for proline and deletion mutants of the PFD [27]. For each mutation, at least 24 individual transformants were assayed for [Het-s] incompatibility function and for their ability to infect a [Het-s*] strain. In this experimental setting, transformants express the transgene from ectopic integration sites and in multiple copies. This approach allows a rough categorization of the mutants. Results are presented in Figure 2. In most cases and as previously observed, the number of transformants able to infect a [Het-s*] strain is equivalent or higher than those producing a barrage reaction to [Het-S] [27]. This is likely due to the fact, that higher prion titers are required to produce an incompatibility reaction than to infect a [Het-s*] strain.

We have categorized the mutants into three functional classes: mutations that lead to percentages of active transformants in the range of wild-type (>60% of active transformants), those significantly reducing the number of transformants with [Het-s] activity (>30%and <60%) and those strongly affecting the number of active transformants (<30%). The majority of the mutations (18/32) did not significantly affect [Het-s] activity in this assay. Nine mutants were slightly affected and five were strongly affected. The latter corresponded to two group of mutants, the mutants of the glycine residue at the sharp turn in position 17 (G242A/G278A) and the aromatic F286 and W287 residues in the C-terminal loop. The Ala replacement of the strongly conserved Tyr residue ending the second rung (Y281) led also to a marked decrease in the number of active transformants. The other mutations having a lesser effect on activity were the mutation of the Asn pair forming the first asparagines ladder (N226/N262, position 1). Mutation of the second Asn pair (N243/N279, position 18) had no effect in agreement with the lower conservation of this second N-pair. In general, mutation of the core hydrophobic residues did not reduce activity significantly with the exceptions of I231A and I277A. Also the Ala replacement at positions E234, K270 and R236 affected the prion function. These residues are involved in the second and third salt bridges in the HET-s fold. Mutations in the residues forming the first salt bridge (K229A/E265A, position 4) had no detectable effect on function in this assay. The variant Q240A showed a reduced activity. Q240 is located close to W287 in the structure [28] and it might be that Q240A also affects this pocket region. Indeed, solid state NMR studies of the variants at the C-terminus results in chemical shift changes of Q240 (as discussed below and Figure S3 and S4). The D288 residue is also located in that spatial neighborhood. The mutational approach points to an instrumental role of this region in prion propagation although this region is not part of the β-solenoid core.

Based on this first series of mutants, we generated a second set of mutants to specifically test several hypotheses. First, because our results suggest that the salt bridges are not essential for activity, we generated a triple mutant that leads to inactivation of all three salt bridges (E234A, E236A, E265A) and found it to be active (Figure 2). In respect to this observation it is noteworthy that, there appears to be an overall conservation of the salt-bridges at position 4 and 9 of the repeat motif, but some HET-S orthologs such as *Cg_HET-S* lack these salt bridges completely (Figure 1B). Next, we have generated a double mutant with both N ladders replaced (N226A and N243A). This mutant lacked activity suggesting that at least one N-ladder is required for function and thus providing evidence for a functional role also for the second N-ladder. In both rungs there is a conservation of an Arg residue (R238/R274, position 13). Yet, single Ala replacements at these positions showed normal function. In a R238A/R274A double variant however the prion function is affected. This result could explain conservation at that position. Finally, we reasoned that if a reduced activity of I231A is caused by a reduction of the hydrophobicity of the core, one might be able to introduce a compensatory mutation increasing hydrophobicity elsewhere in the core. We thus changed the A228 residue of the core by a larger residue. We generated a I231A/A228V double variant and found that this double mutant recovered wild-type like activity.

We concluded that in this multicopy assay, the function of HET-s appears to be very robust with very few mutations having a very strong effect on prion activity, namely G242A and G278A in the turns after the triangular core and F286A and W287A in the C-terminal pocket. Double mutants could also demonstrate the functional importance of other elements such as the N-ladders, the hydrophobic core (i.e. I231), and the Arg row at position 238/274.

### Detailed analysis of selected mutants assayed as single copy integrants

The multicopy assay allows an initial functional characterization of the mutants but does not allow for a detailed and sensitive analysis of phenotypic differences. We have thus selected a number of mutants for a more detailed functional characterization as single copy integrants. In order to have more subtle and sensitive assays for prion function, we have re-introduced the mutant allele at the resident *het-s* locus. We chose to further analyze a subset of seven single mutants N226A, Q240A, E272A, K284A, F286, W287, D288A and the R238A/R274A and T233A/S273A double mutants and the E234A/R236A/E265A triple mutant. Q240A, K284A, F286A, W287A and D288A were chosen to investigate the role of the C-terminal loop region. The triple E234A/R236A/E265A mutant was chosen to analyze in more detail the effect of the elimination of all three salt-brigdes. R238A/R274A and T233A/S273A double mutants were included to analyze respectively the role of the conserved Arg residues close to the loop and the role of the partner hydroxyl residues in the core.

**Figure 3:**
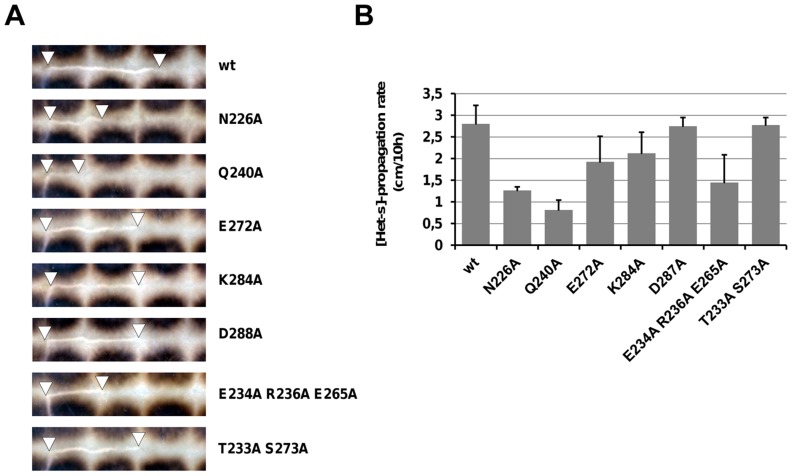
Propagation rate of [Het-s] in single copy integrants of alanine mutants. **A.** For wt and each mutant, the rate of [Het-s] propagation was measured in the experimental setting in which the length of the barrage line gives the distance of [Het-s] spreading in a period of 10 hours. **B.** Mean propagation distance in at least 4 experiments with standard deviation.

Strains with the corresponding gene replacements were generated and tested for [Het-s] function. The three mutants F286A, W287A and R238A/R274A led to a null phenotype as single copy integrants. This result confirms the functional importance of the aromatic residues of the C-terminal pocket and also stresses the functional importance of the conserved Arg residues at position 13 of each repeat. The remaining mutants were able to express the [Het-s] phenotype (*ie*. infect a [Het-s*] strain and produce a barrage reaction to *het-S*) and were tested for three other criteria of prion formation and propagation efficacy. First, we determined the spontaneous prion formation rate (Table 1). Secondly, we measured the rate of spreading of the prion infection in the mycelium, reflecting prion propagation efficiency (Figure 3). This propagation rate is measured in an experimental setting where the length of the barrage line corresponds to the distance of prion spreading in a given period of time [54] (Figure S2). Third, the rate of spore-killing in the sexual cycle (a measure of prion maintenance during the sexual cycle) was determined [24], (Table 2). These three criteria have been compared previously between wt and a *ΔPaHsp104* mutant. All three were found to be diminished in this mutant background [55].

**Table 1:** Spontaneous [Het-s] prion formation rate for alanine mutants of HET-s.

Strain	[Het-s]/total tested	% of prion formation	p-value (significance of difference to wt)**
WT	13/600	2.1%	-
F286A	0*	n.a	
W287A	0*	n.a	
R234A R274A	0*	n.a	
N226A	3/500	0.6%	<0.05
Q240A	1/400	0.3%	<0.02
E272A	15/400	3.7%	n.s.
K284A	0/700	0	<0.0001
D288A	21/500	4.2%	<0.05
E234A R236A E265A	6/500	1.2%	n.s.
T233A S273A	24/500	4.8%	<0.02

*these mutants are unable to propagate [Het-s] even after infection by wt [Het-s].

**p-value is calculated in a two-tailed Fisher's test, n.s. non significant.

**Table 2:** Spore-killing activity of selected *het-s* mutants in a cross a with *het-S* male parent.

maternal parent in cross	2-spored asci/total counted asci	spore-killing (%)	p-value (significance of difference to wt)*
*het-S*	0/272	0	<0.0001
*het-s* wt	445/632	70.4	-
N226A	7/664	1	<0.0001
Q240A	0/425	0	<0.0001
E272A	328/746	44	<0.0001
K284A	31/506	5.8	<0.0001
D288A	190/509	37.3	<0.0001
T233A/S273A	311/836	37.2	<0.0001
E234A/R236A/E265A	94/654	14.4	<0.0001

*p-value is calculated in a two-tailed Fisher's test.

In these assays, all mutants were affected for at least one of the measured criteria. There was in general a good correlation between the effect of the mutations on propagation rate and spore killing activity (Figure 3 and Table 2). Quantitatively the effects of the mutations were however stronger on spore-killing rates than on propagation rates. For instance N226A and Q240A were affected both in propagation rate and spore-killing activity. While E272A, D288A and T233A/S273A showed close to wt propagation and killing rates. K284A was an exception as this mutation affected spore-killing rates although the propagation rate was close to wt. The E234A/R236A/E265A triple mutant lacking all three salt bridges was significantly affected in both assays, suggesting that the salt bridges participate in [Het-s] function although the effect of the mutation went unnoticed in the more basic multicopy assay (Figure 2). When spontaneous prion formation rates were measured, it appeared that N226A and K284A had significantly reduced spontaneous prion formation rate (Table 1). For K284A, 700 strains were tested but none had spontaneously acquired [Het-s]. Interestingly, we measured an increase in spontaneous prion formation rate in D288A and the T233A S273A double mutant. These mutants represent to our knowledge the first HET-s mutants with enhanced prion behavior (Table 1).

### Solid-state NMR of amyloid fibrils of HET-s(218-289) variants

In order to get insights into the structural consequences of the Ala replacements, 2D ^13^C-^13^C dipolar assisted rotational resonance (DARR) solid state NMR spectra of amyloid fibrils of the ^15^N,^13^C-labeled HET-s(218-289) variants K229A, I231A, V239A, Q240A, L241A, N262A, V264A, V267A, E272A, S273A, R274A, I277A, G278A, N279A, F286A, D288A and the double variant F286A/W287A were recorded and compared with the corresponding spectrum of ^15^N,^13^C-labeled wild-type HET-s(218-289) amyloid fibrils [27], [56]. Since the chemical shift is a highly sensitive probe of the electronic surrounding structure, the measurement of the 2D ^13^C-^13^C DARR spectrum can be regarded as a fingerprint of the 3D structure. Thus, a comparison of the DARR spectrum of wild-type and Ala-variant fibrils enables a straight forward analysis of the preservation or alteration of the β-solenoid fold (Figure 1A). This atomic-resolution analysis is of particular importance in the establishment of a structure-activity relationship of amyloids because many amyloidogenic systems are prone to polymorphism and thus a secondary structure analyses or structural comparisons conducted at the mesoscopic level may not be not sufficient to reveal structural preservation or alterations.

**Figure 4:**
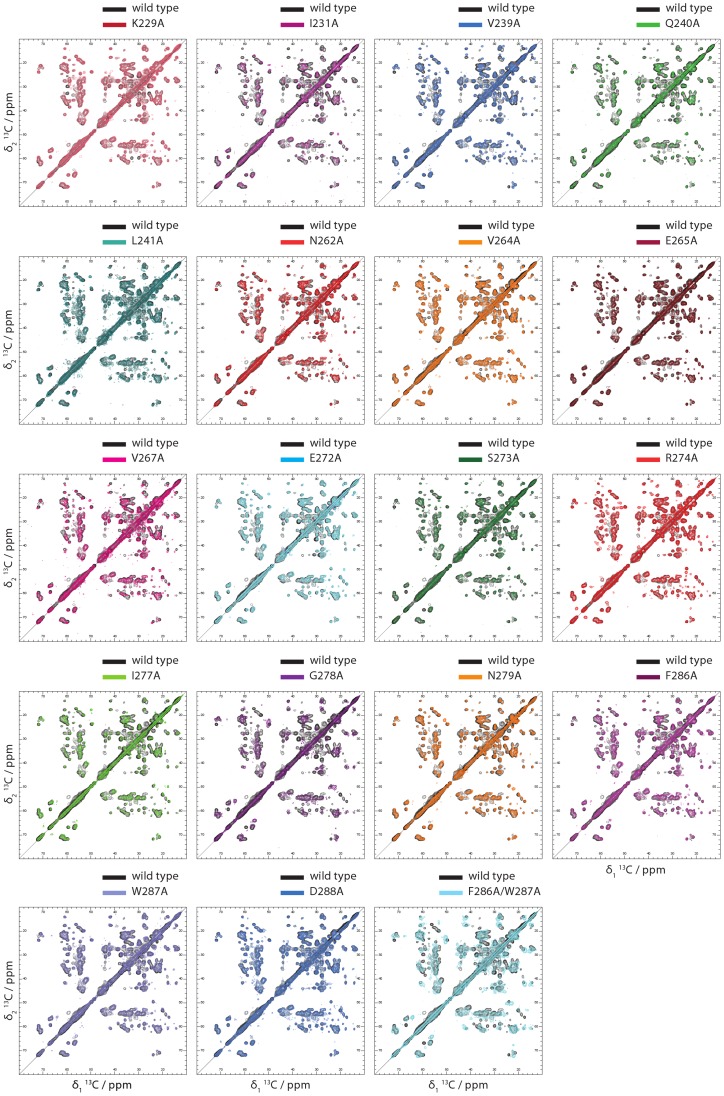
2D Solid state NMR spectra of Ala variants of HET-s(218–289) amyloid fibrils. The 2D DARR solid state NMR spectra of ^15^N,^13^C-labeled Ala variants of HET-S(218–289) amyloid fibrils are shown on top of the corresponding spectrum of wild-type HET-s(218–289) amyloid fibrils, the latter which contour lines are color-coded black. The spectra are labelled according to the amino acid replacement. The close resemblance between the variant and the wild-type spectra indicates the conservation of the β-solenoid fold in the variants with the exception for G278A and the double variant F286A/W287A. In Figure S3 the same spectra are shown including the assignment of wild-type HET-s(218–289) amyloid fibrils [29].

As shown in Figure 4, the 2D DARR spectra of K229A, I231A, V239, Q240A, L241A, N262A, V264A, E265A, V267A, E272A, S273A, R274A, I277A, E279A, F286A, W288A, and D288A closely resemble the wild-type spectrum. This indicates that the overall structure of the listed variants is conserved. A detailed inspection of the individual spectra shows in general, small chemical shift differences of residues close in space to the replaced amino acid side chain (in addition to the lack of the signal of the replaced side chain), which is attributed to local structural changes. For example, in the 2D DARR spectrum of the N262A variant, cross peak changes of the spatially close residues I231, T261, S263, V264,and I277 are observed (Figure 4, Figure S3, Figure S4, Figure 1A). In the case of E265A located at the surface, no significant chemical shift changes are evident (Figure 4, Figure S3, Figure S4, Figure 1A). Similar findings are observed for all the other Ala variants (but G278A, and the double variant F286A/W287A, see below) as discussed in details in the figure caption of Figure S3. In the case of I231A, a substantial amount of small local chemical shift perturbations are observed for A228, K229, D230, T233, V239, L241, T266, V267, V268, V275 and I277 as well as for the more remote residues E235, V244, A247, A248, K270, R274, and L276, hinting that I231 is a structurally important hydrophobic core residue. In addition to these local chemical shift alterations, (slight) perturbations of chemical shifts of I277 are found for many Ala variants (i.e. I231A, Q240A, N262A, V264A, V267A, R274A, F286A, W287A) indicating that I277, which is the second Ile side chain in the center of the core of the β-solenoid structure (Figure 1A), responses sensitively to slight structural alterations.

In striking contrast to all the variants discussed, the 2D DARR spectrum of the ^15^N,^13^C-labeled variant G278A is reproducibly distinct from the corresponding spectrum of wild-type HET-s(218–289) amyloids (Figure 4). The spectrum appears to be less well dispersed and the cross peaks are broad when compared to wild-type HET-s(218–289) amyloids. The spectrum is actually reminiscent to the spectrum of the non-infectious HET-s(218–289) amyloid grown at pH 3 which was also found to be less dispersed and to have broad cross peaks [42]. Hence, it is concluded that the G278A variant does form a distinct amyloid structure, which may serve as an explanation of the lack of a prion phenotype (Figure 2; and see below). The nature of the misfolding of the G278A variant may be that the Gly residue is key for the arc between the two β-strands β4a and β4b. In addition, the F286A/W287A double variant shows a 2D DARR NMR spectrum quite different from the wild-type DARR spectrum (Figure 4). There are major chemical shift perturbations spread over the β-solenoid structure (i.e. I231, S273, E235, K229, T233, N262, R238, A247, V264, V244, I277, and Q240), making it difficult to assess whether the double variant forms the β-solenoid fold (Figure 4).

In conclusion, the comparative analysis of the solid state NMR spectra suggests that all the single Ala variants with the exception of G278A form the HET-s prion β-solenoid fold. With the exception of G278A, all the single Ala variants can therefore be used to explore the detailed structure-infectivity relationship of the HET-s prion.

### Stability measurements of amyloid fibrils of HET-s(218–289) variants

**Figure 5:**
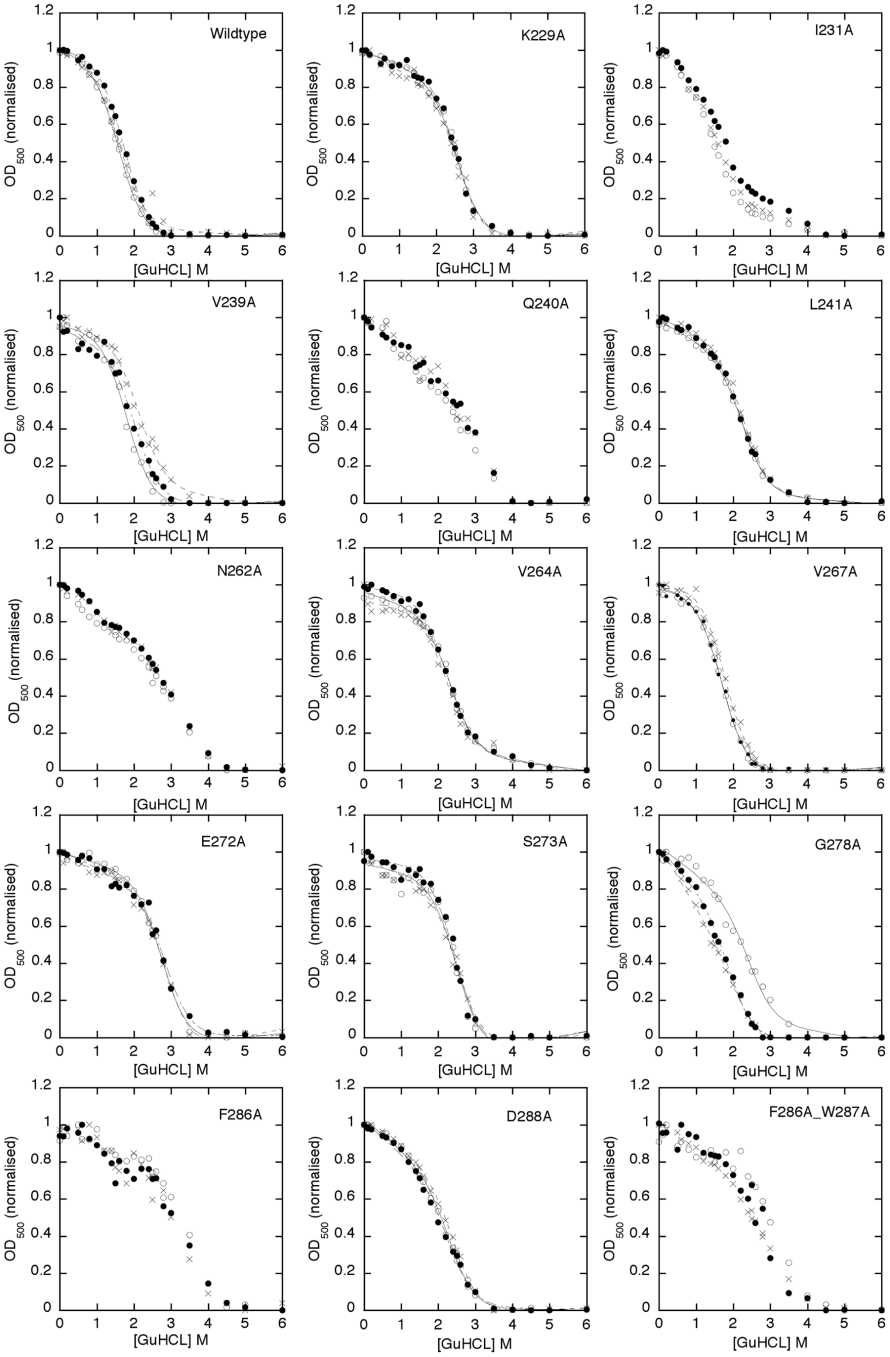
GuHCl unfolding measurements of HET-s(218–289) amyloid fibrils and Ala variants thereof. The GuHCl denaturation curve of amyloid fibrils of HET-s(218–289) and Ala variants (as indicated) were measured by the OD_500_ (y-axis) at various GuHCl concentration (x-axis) after incubation in the corresponding GuHCl buffers for one day. The change of the OD_500_ value is indicative of the loss of large aggregated protein species. Three samples each were incubated and the individual measurements are highlighted by asterisks, by small dots, or by open circles, respectively. Individual successful fits following the concept by Santoro and Bolen to study protein folding and unfolding [61] are shown by black lines and the calculated change in Gibbs energy (ΔG) with its standard deviation from the three measurements are listed in Table 3.

One of the consequences of a side chain replacement with Ala may be a change in the stability of the protein fold, which may influence function [57], [58], [59], [60]. Applied to HET-s this would mean that a change of the stability of the β-solenoid may alter heterokaryon incompatibility, spontaneous prion formation, prion propagation and spore killing (Figure 2, Figure 3, Tables 1 and 2). In order to get insights into the individual contribution of the amino acid side chains to the stability of the HET-s prion, Ala variants K229A, I231A, V239A, Q240A, L241A, N262A, V264A, V267A, E272A, S273A, G278A, F286A, D288A and F286A/W287A were measured by a fibril denaturation assay using GuHCl (Figure 5 and Table 3) following the concept by Santoro and Bolen to study protein folding and unfolding [61]. However, here we studied only the fibril denaturation process. This was monitored by measuring the light scattering at 500 nm (OD_500_) [62] versus GuHCl concentration (Figure 5) detecting fibril disassembly. Since the β-solenoid amyloid structure is composed of many inter-molecular interactions it is assumed that disassembly and unfolding occur together and that this detection method measures thus both fibril disassembly and β-solenoid unfolding. Such a fibril denaturation reaction mechanism can be of complex nature but here a simplified two-state process was assumed for the quantitative analysis. HET-s fibril denaturation studies have been performed previously using Trp fluorescence as probe for fibril denaturation [41], because the unique Trp residue (W286) is located in the C-terminal loop rather than in the actual core, we favored the use of light scattering because it might constitute a better probe for the overall unfolding of the fibril since Trp fluorescence might be affected by local unfolding of the loop.

**Table 3:** Gibbs free unfolding energy of HET-s(218–289) amyloid fibrils.

Variant	ΔG(a) (kcal/mol)	ΔΔG (kcal/mol)	m-value	Reproducibility and denaturation curve shape when compared to wt	*In vivo* [Het-s] Property ([Het-s] activity/propagation rate/spore killing (Figure 2; Tables 1–2)
WT	2.9±0.2		wt	y, wt	
K229A	4.4±0.0	1.5±0.2	wt	y, wt-like	wt-like
I231A	n. d.	n. d.	not wt	y, no plateau at low GuHCl conc.	[Het-s] reduced
V239A	3.9±0.5	1±0.5	wt	y	wt-like
Q240A	n. d.	n. d.	not wt	n, no plateau at low GuHCl conc., not a two-state process	[Het-s] reduced/reduced propagation and spore killing
L241A	3.9±0.1	1.0±0.3	wt	y, wt-like	wt-like
N262A	n. d.	n. d.	not wt	y, not a two-state process	[Het-s] reduced
V264A	4.0±0.2	1.0±0.3	wt	y, wt-like	wt-like
V267A	2.9±0.1	0.2±0.3	wt	y, wt-like	wt-like
E272A	4.8±0.1	1.9±0.3	wt	y, wt-like	wt-like/reduced propagation
S273A	4.3±0.1	1.4±0.2	wt	y, wt-like	wt-like
G278A	2.5±0.3(b) 1.3±0.1(b)	-0.8±0.4	not-wt	n, no plateau at low GuHCl conc.	[Het-s] strongly reduced
F286A	n.d	n.d	not wt	y, not a two-state process	[Het-s] strongly reduced
D288A	4.1±0.1	1.2±0.3	not wt	y, wt-like	[Het-s] reduced/enhanced prion formation
F286A/W287A	n.d	n.d	not wt	y, not a two-state process	n.d.

(a) The ΔG value was obtained using the m-value of the wt unless stated in the fourth column that the curve fitting was improved with a free m-value different from the corresponding wt one.

(b) The ΔG value was obtained using a free m-value. Two different denaturation curves were obtained and their individual ΔG values are listed.

The individual denaturation curves in triplicates show a sigmoidal behavior (Figure 5). [61]. For most of the variants (i.e. wild-type, K229A, I231A, V239A, Q240A, L241A, N262A, V264A, V267A, E272A, S273A, F286A, D288A) the measurements appear to be reproducible. Only for G278A and possibly the double variant F286A/W287A a lack of reproducibility is observed (Figure 5). The lack of reproducibility is attributed to the altered, possibly polymorphic structure as evidenced by the solid state NMR spectra (Figure 4). A further qualitative inspection of Figure 5 suggests that the variants K229A, V239A, L241A, V264A, V267A, E272A, S273A, and D288A (labeled group 1 in the following) show sigmoidal denaturation curves similar to wild-type, while the denaturation curves of the variants I231A, Q240A, N262A, G278A, F286A (group 2), and the double mutant F286A/W287A have a different form and properties and are sometimes less reproducible (as just stated) (see also Table 3, fifth column).

The two-state analysis of the denaturation curves yields two parameters for the unfolding transition: the *m-value* and the Gibbs free energy ΔG. The *m*-value is the steepness of the transition and is generally thought of as a measure of the change in solvent accessible surface area upon unfolding and reflects how cooperative the unfolding transition is [63], [64], [65]. The change in Gibbs free energy (ΔΔG) is a measure of the contribution of the amino acid side chain to the protein unfolding energy (given that the structures of the folded and unfolded states as well as the unfolding pathway are otherwise unperturbed). The variants of group 1 can all be fitted with a two-state model, most of them with an unchanged *m*-value (i.e. the *m*-value of the wildtype HET-s(218–289) prion). All the group 1 variants had an unaltered ΔΔG value or were up to 2 kcal/mol more stable (Table 3). The positive ΔΔG value for K229A of 1.5 kcal/mol indicates that the salt bridge between K229 and E265 does not play a favorable stability effect on the β-solenoid structure. Similarly, the replacement E272A (removing the E272-R236 salt bridge) results in a positive stability effect of approximately 2 kcal/mol. The amino acid replacements in the hydrophobic core at position V239, L241, V264 also had a 1 kcal/mol positive effect on stability V267 had no effect. This is in line with the *in vivo* data showing that none of these variants were affected for the prion phenotype (Figure 2). In combination with the solid state NMR spectra (Figure 4 and see above), these unfolding measurements indicate that the Ala variants of group 1 are composed of the same β-solenoid 3D structure and the amino acid side chain replacement had no negative effect on its unfolding transition.

In contrast, the GuHCl denaturation curves of group 2 variants (Figure 5) are distinct from wild-type showing a flattened slope (lower *m*-value) (i.e. I231A, Q240A, and N262A) indicative of a decrease in unfolding cooperativity or a change of the surface. The latter potential explanation finds support in the surface location of the Q240. Some replacements comprise a complex unfolding transition exemplified by an immediate loss of light scattering at low GuHCl concentrations (i.e. I231A, Q240A, N262A, and F286A) or poor reproducibility (i.e. G278A and F286A/W287A), thus for these variants a Gibbs free energy calculation has not been considered (see Table 3). It is worth mentioning, that the less reproducible nature of the denaturation curves of G278A and F286A/W87A is reflected in the less well defined solid state NMR spectra (Figure 4). Together the data strengthen the notion that these two variants are not able to fold properly into the β-solenoid structure. The effect on the denaturation curve of N262A is attributed to the loss of the conserved Asn ladder. The I231A in the hydrophobic core replacement appears to influence the stability and unfolding pathway (Table 3). The NMR analyses also indicated an important structural role of this residue as the I231A replacement leads to a substantial amount of chemical shift perturbations in the β-solenoid fold (Figure 4, Figure S3 and S4). Most interestingly, with the exception of D288A there appears to be a perfect correlation between the decrease in prion phenotype formation *in vivo* determined by the heterokaryon incompatibility assay (Figure 2) and the change in the denaturation curve profile (Table 3).

### The F286A and W287A variants of C-terminal pocket are null alleles but the corresponding proteins form infectious fibrils *in vitro*

**Figure 6:**
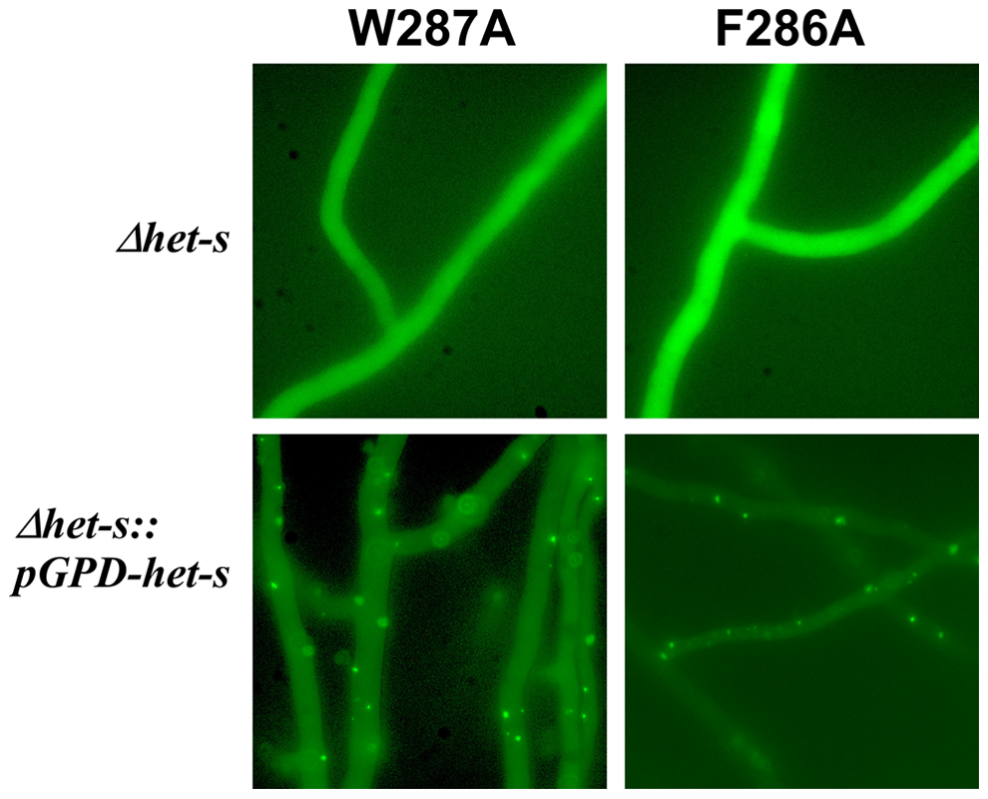
Expression of a HET-s-GFP F286A and W287A fusion proteins in *P. anserine*. HET-s-GFP F286A and W287A were expressed in *Δhet-s P. anserina* strain or the same strain over-expressing wild-type HET-s. Note that both mutants are unable to form dot-like aggregates in *Δhet-s* but can be incorporated in dot-like aggregates in the presence of wild-type HET-s.

The F286A and W287A mutants affecting the C-terminal semi-hydrophobic pocket stand out in virtue for their drastic effect on [Het-s] activity although the corresponding residues reside outside the rigid β-solenoid core and do not alter the β-solenoid structure significantly (Figure 4). Only the double variant F286A/W287A may be unable to form the β-solenoid structure as suggested by the solid state NMR studies (Figure 4) and the GuHCl denaturation curves (Table 3, Figure 5). Moreover, two further mutants in the same region K284A and D288A also modulate prion behavior in a subtle way as the first mutation decreases prion formation while the other in fact increases the prion formation rate (Table 2). However, the D288A variant shows a wild-type-like solid state NMR spectrum and is ca. 1.2 kcal/mol more stable than wild-type, having otherwise a wild-type-like GuHCl denaturation curve (Figure 4 and 5). We chose to further analyze the mutants in that region for their ability to form aggregates *in vivo* as GFP fusion proteins. We found that neither F286A nor W287A led to the formation of dot-like aggregates *in vivo*, however when the mutant allele as GFP fusion proteins were introduced in a strain expressing wild-type HET-s (Figure 6), the mutant alleles were found to form dot-like aggregates presumably by being integrated into wild-type aggregates, a situation already described for the K284P mutant residing in the same conserved C-terminal loop region [27].

We also tested infectivity of the variants F286A and W287A fibrils generated *in vitro* and found them to be infectious (Table 4). The level of infectivity of the recombinant fibrils was at least as elevated as for wild-type fibrils. Infectivity could be detected in the same level of dilutions as for wild-type fibrils. These results indicate that once the amyloid fold is acquired, these mutants display wild-type seeding activity *in vivo*.

**Table 4:** Infectivity of recombinant HET-s(218-289) F286A and W287 fibrils.

Amount of protein	[Het-s]-infected strains over total tested strains
no protein	1/96
HET-s(218–289) wt	
250 µM	63/96
50 µM	15/72
10 µM	1/48
2 µM	0/24
HET-s(218–289) F286A	
250 µM	69/96
50 µM	25/72
10 µM	5/48
2 µM	0/24
HET-s(218–289) W287A	
250 µM	68/96
50 µM	12/72
10 µM	2/48
2 µM	0/24

## Discussion

The [Het-s] prion of the filamentous fungus *Podospora anserina* represents an example of a functional amyloid. The amyloid fold serves as an activation trigger for inducing the transformation of HET-S into a toxin [30]. We have conducted a mutational analysis of HET-s PFD to assess the role of specific residues on prion function and propagation, as well as amyloid structure and stability. It is observed here that only very few Ala point mutations are able to abolish prion function, alter significantly the β-solenoid structure and reduce fibril stability. Most mutants retain partial function indicative of a robust prion. These studies also revealed a previously unsuspected role for the C-terminal pocket region in prion function.

### Structure-function of the HET-s fold

The determination of the HET-s β-solenoid structure exposed the existence of a number of structural elements defining this fold, such as the hydrophobic core, the asparagines ladders, the sharp turns connecting the β-strands termed arches [66], the salt bridges, the internal hydrogen bond between the Thr and Ser residing in different β-strand layers and the C-terminal loop folding back onto the core [28], [29]. The functional importance of the individual β-strands was documented before in a mutational approach involving proline substitutions [27]. Here, it is shown that to various degrees, the other described structural determinants also participate in prion function.

Ala replacement of the strictly conserved G278, leads to a null prion phenotype and this variant is unable to form the β-solenoid structure as evidenced by the solid state NMR studies. This effect is very likely due to the importance of the glycine residue in the arc between β-strands β4a and β4b for the β-solenoid fold (Figure 1). Ala replacements in the asparagine ladder affected function *in vivo* and the N262A variant shows an altered denaturation curve. The hydrophobic core residues I231 and I277 appear to be important for prion infectivity (Figure 2) and in the case of I231A also for β-solenoid unfolding and structure (Table 3, Figure 4). Also, the substitution of the highly conserved Thr/Ser pair leads to a detectable alteration of prion function but only in the spore-killing assay, while the S273A variant is 1.4 kcal/mol more stable. For the prion function *in vivo*, the solvent exposed salt bridges are important determinants, but their contribution to the β-solenoid stability is negative (i.e. E272A is 1.9 kcal/mol more stable than wild-type). Sequence comparisons shows a preference for a positively charged residue in position 13 of each repeat (R238 and R274 in HET-s) (Figure 1). The functional significance of these conserved Arg residues is not directly evident. While the single Ala replacement did not change the prion activity, the R238A/R274A double mutant leads to a null phenotype thus providing a functional justification for the sequence conservation. A possible explanation of the role of these positively charged residues could be an interaction with the C-terminal loop and in particular the penultimate conserved Asp residue. In support of this hypothesis, is the fact that the two sequences that are exceptions to the conservation of positively charged residues at these positions also lack the C-terminal glycine-rich loop like *Fo_CL52-2* and *Nh_mpVI-1*, (Figure 1B).

In the presented Ala mutagenesis scan, two mutants were found for which spontaneous prion formation rate was increased rather than decreased, the D288A mutant and the T233A/S273A double mutant. This would indicate that the HET-s PFD does not have an optimal behavior in terms of prion formation rates. It might be that the increased prion formation rate of D288A is due to the higher stability of the variant (i.e. ΔΔG = +1.2 kcal/mol, Table 3) or/and a modification of the properties of the C-terminal loop which is functionally important for a prion phenotype (see below). The T233A/S273A double mutation increases the overall hydrophobicity of the core. Possibly, this increase in hydrophobicity favors a hydrophobic collapse and subsequent prion nucleation and/or stabilizes the β-solenoid fold since already the amyloid of S273A alone is 1.4 kcal/mol more stable (Table 3). Interestingly, the D288 and the S273 residues (and to a lesser extend T233) are conserved positions (Figure 1B). Thus, one may hypothesize that conservation at these positions reflects the need to reduce spontaneous β-solenoid folding because a too strong propensity for spontaneous folding might lead to uncontrolled activation of the HET-S pore-forming toxin. Similarly, the fact that several replacements increase rather than decrease stability also suggests that the HET-s PFD sequence is not optimized for highest possible stability. The theoretical model of prion propagation by the Weissman group [67] and others [68], [69] highlights that for the prion propagation, amyloid fibril fragmentation is necessary. Thus, highest possible amyloid stability may not be compatible with the function of the HET-s fold.

### Modulating prion formation by a short sequence stretch located outside of the cross-β core

One finding of this study is the detection of the functional importance of a 7 amino acid residues C-terminal loop starting at the end of β-strand β4b and ending at the C-terminus of the protein (GKGFWDN, residue 283 to 289). The only previous indication of the functional importance of this region came for the observation that the K284P mutation affects HET-s aggregate formation *in vivo* [27]. Structurally, this region forms a semi-hydrophobic pocket that folds back onto the space delimited by the third and fourth strand of each rung [28]. This part of the structure is less well defined and appears to be more dynamic than the core region. The individual mutations of the two aromatic residues F286A and W287A have a very strong effect on activity. Actually, these two mutations are the most severe ones in our set of 32 mutants together with the mutations of the Gly in the arc positions (i.e. G242/G278). In addition, the mutations of the conserved charged residues in that region K284A, D288A (and the structurally nearby Q240A) also affect function. As noted above, the D288A mutation produced an unexpected effect on prion function as this mutation increased spontaneous [Het-s] prion formation. This is the first mutation found to increase [Het-s] prion formation. Of note however is that other criteria such as the spore-killing activity are in fact slightly reduced in that mutant.

A recent molecular dynamics simulation study of the HET-s(218–289) β-solenoid suggested that the F286A and W287A mutations reduce flexibility at the fibril ends and that this flexibility is an important determinant for prion fibril infectivity [70]. However, F286A and W287A are able to form fibrils *in vitro* that display the same levels of infectivity as wild-type fibrils suggesting that mutation of the aromatic residues does not critically affect templating activity and the 3D structure of the β-solenoid fold. Alternatively, the hydrophobic nature of F286 and W287 may be important for an initial non specific hydrophobic interaction of the incoming molecule with the prion template. This hypothesis is supported by the solid state NMR spectra of both variants indicating no significant structural changes of the β-solenoid fold. Rather it appears that this region might be required for the prion formation process without actually being part of the cross-β part of the fold. These interpretation illustrates that amyloid folding can be dramatically modulated in a functionally relevant way by a sequence stretch that is not actually part of the cross-β structure *per se*. Strikingly, this part of the HET-s PFD sequence turns out to be the region of the protein with the highest level of sequence conservation. While, the repeat region can accommodate a significant level of divergence and still retain the ability to adopt the β-solenoid fold, the ability for this short C-terminal region to act possibly as a folding inducer of the repeat region appears highly constrained at the sequence level. The β-solenoid forming region of HET-s/HET-S is thus located between the HeLo domain which can have a prion inhibitory function and the C-terminal pocket region which acts as a prion promoting region [26]. These results revealing the existence of a short amyloid folding modulator stretch that is not directly involved in the cross-β core could be of general importance in the identification of sequence determinants for relevant amyloid formation and in efforts to control amyloid aggregation in disease related systems.

### Level of complexity of the β-solenoid fold

Since many proteins are able to aggregate into amyloid-like entities and since the inter-molecular β-sheet formation with its many hydrogen bonds is an essential determinant of the cross-β-sheet motif, it has been suggested that the formation of amyloid fibrils is mainly a generic property of the polypeptide backbone and that the side chains play a minor role [71]. On the contrary, amyloid aggregation is highly amino-acid sequence specific as demonstrated by the intermolecular side chain interactions observed in the crystal structures [72], [73], [74] and the essential involvement of side chain interactions in the aggregation process as evidenced for example by the experimentally-derived scale of amino-acid aggregation-propensities (ranging from the aggregation-prone hydrophobic residues to the aggregation-interfering charged side chain [75], [76], [77]). These observations accompanied with the predictive power of several algorithms for the cross-β aggregation propensity of polypeptide sequences [78], [79], [80] suggests that the cross-β state is less complex than most structures of soluble proteins, indicating that the complexity of the cross-β fold may fall somewhere between a secondary and a tertiary structure [2].

This notion of intermediate folding complexity of amyloids is now supported by the stability measurements of the HET-s Ala variants. Indeed, our study reveals both the absence of destabilizing effect for many replacements but also the critical importance of certain key residues. While the loss of side chain hydrogen bonds, salt bridges and hydrophobic interactions in soluble proteins yields in general a ΔΔG loss of ∼−2 kcal/mol [58], [59], [60], in the case of the HET-s prion, the ΔΔG for the group 1 variants with K229A and E272A deleting a salt bridge, S273A getting rid of a side chain hydrogen bond, and V239A, L241A, V264A, and V267A reducing the hydrophobicity of the core, is either insignificant or with a positive value of up to +2 kcal/mol (Table 3). In particular, hydrophobic core residue replacements have little effect (+0.6 kcal/mol in average; Table 3) compared to the average ΔΔG of ∼−2 kcal/mol predicted from data of soluble proteins. Only a few side chains appear to be crucial for the formation of the β-solenoid structure (such as for instance I231 and N262). Side chain replacements of such residues lead to *in vivo* effects but also to noticeable changes of the GuHCl denaturation curve. Thus, it appears that alteration of the β-solenoid structure can be achieved either by Pro insertions [27] having a drastic consequences on the backbone structure or by replacing side chains of a limited number of key residues that appear critical for directing the peptide backbone into the β-solenoid fold.

## Materials and Methods

### Strains, plasmids and media

*P. anserina* strains used in this study were wild-type *het-s*, *het-S*, *Δhet-s* strains and the *ΔPaHsp104* strain [55]. Growth medium for barrage assays and prion transmission assays was standard corn meal agar DO medium. The *Δhet-s* strain was constructed by inserting the *nat1* cassette from the pAPI508 plasmid [81] in place of the *het-s* ORF. The *nat1* cassette was amplified with oligonucleotides 5′ CTTCCCTTCCACTTCTTCACAC 3′ and 5′ ATCCTAGATGACTTAAGACGACAGG 3′. The sequences upstram and downstream of the *het-s* ORF were amplified respectiviely with oligonucleotide pairs 5′ AAGCTTTTCGAATTGGTCTCTCAG 3′ and 5′GGGCAGTTTGAGGGGAAAGCGAAG 3′ and 5′ GGGACTAGTACCCTCCAGCAAGGATAGC 3′ and 5′ GCGGCCGCCATGGGCACTGCATCTGGG 3′. The fragments were ligated to create the *het-s::nat-1* cassette cloned as a *Hind*III-*Nco*I fragment in a pSK plasmid. This cassette was used to transform a *het-s ΔPaKu70* strain [81]. Nourseothricine resistant transformants compatible with a *het-S* tester were selected. Inactivation of *het-s* was verified by PCR using oligonucleotides 5′ CGACGATCACAGCTATAGCGTGGTG 3′ and 5′ ATCCGGCTTCCCTGGACCTGCTTC 3′. The strain was then backcrossed once to a *het-s* wild-type strains and *Δhet-s* (Nour^R^ and *het-S* compatible) *Δhet-s ΔKu70* strains (*Nour^R^, Phleo^R^* and *het-S* compatible) were selected in the progeny.

### Prion propagation, prion formation and incompatibility assays

Methods for determination of incompatibility phenotypes, prion formation and prion propagation were as previously described [54]. In brief, incompatibility phenotypes were determined by confronting strains of solid corn meal agar medium to [Het-s] and [Het-S] tester strains and visualizing the formation of barrages (abnormal contact lines forming upon confrontation of incompatible strains). [Het-s] prion propagation was assayed as the ability to transmit the [Het-s] prion from a [Het-s]-donor strain to a [Het-s*] prion-free tester strain after confrontation on solid medium. Prion formation rates of [Het-s] were determined by measuring the fraction of [Het-s*] or subcultures that spontaneously acquired the prion phenotype after 5 days of growth at 26°C.

### Microscopy

For fluorescence microscopy, synthetic medium containing 2% (wt/vol) agarose was poured as two 10 ml layers of medium. *P. anserina* hyphae were inoculated on this medium and cultivated for 16 to 24 h at 26°C. The top layer of the medium was then cut out and the mycelium was examined with a Leica DMRXA microscope equipped with a Micromax CCD (Princeton Instruments) controlled by the Metamorph 5.06 software (Roper Scientific). The microscope was fitted with a Leica PL APO 100X immersion lens.

### Protein preparation and fibril formation

HET-s(218–289), HET-s(218–289) Ala variants proteins were expressed in *E. coli* and purified as previously described [27]. Both proteins had a C-terminal 6 histidine tag and expressed as insoluble proteins and purified under denaturing conditions using Qiagen columns. Yields were in the range of 10 mg/L of culture. Proteins were eluted in 6 M GuHCl 50 mM Tris–HCl pH 8, 150 mM NaCl, 200 mM imidazole. Elution buffer was replaced by 175 mM acetic acid by passage on a 5 ml Hitrap column (Amersham). After lyophilisation the samples were stored at −20°C.

The protein variants were dissolved in diluted HCl (45 mM) and the concentration adjusted to 90 µM which was affirmed by UV absorption at 280 nm. The solution was filtered through a 0.2 µM filter and Buffer E (Tris [3 M]; NaCl [1 M] HCl [45 mM] 1/19 V) was added such that the end concentration of NaCl and Tris were 150 and 50 mM respectively. If necessary pH was adjusted to 7.4 using NaOH (4 M). Samples were rotated at 37°C on a rotator at constant speed for at least one week.

### Protein transfection assays

Protein transfection experiments with amyloid fibrils of recombinant HET-s(218–289) variants were performed using a cell disruptor (Fast-prep FP120, Bio101, Qbiogen Inc.). For each test, ∼0.5 cm^3^ of [Het-s*] mycelium grown on solid medium is sheared (run time 30 s, speed 6) in 500 µl of STC50 buffer (0.8 M sorbitol, 50 mM CaCl_2_, 100 mM Tris HCl pH 7.5) and the sonicated HET-s(218–289) amyloids assembled at pH 7 (20 µl at 1 mM) in a 2 ml screw cap tube. The sheared mycelium is then diluted with 600 µl of STC50 buffer and then plated onto DO−0.8 M sorbitol medium and incubated at 26°C until being confluent (7–8 days). Several implants (at least two per mycelium) are checked for the [Het-s] phenotype in barrage tests.

### Denaturation measurements of amyloid fibrils of HET-s(218–289) variants

The fibril containing samples of the various HET-s(218–289) variants were concentrated after centrifugation (20000 g; 10 min) to 500 µM. The fibrils were then diluted in the different GuHCl stock solutions (between 0–8 M in 0.5 M steps) to an end concentration of 20 µM. It is important to note that the fibril samples were added to the GuHCl solution by an Eppendorf pipette with a single extrusion. The samples were incubated at room temperature without shaking for one day or if stated for one week, respectively. The extent of amyloid disassembly was monitored by the measurement of the optical density at 500 nm (OD_500_) on a JASCO V-650 spectrophotometer with quartz cuvettes and an adaptor from Eppendorf. The OD_500_ is believed to be an indirect measure of the amount of protein aggregation. Each experiment has been measured three times starting in part from different sample preparations. The analysis and extraction of the ΔG and m-value was done by the software package MATLAB following the mathematical formula and procedures of Santoro and Bolen [61].

As stated above the samples were incubated before the OD_500_ measurement at the various concentrations of GuHCl for one day in order to reach equilibrium conditions. Longer incubation periods such as one week did either not alter the measurements as demonstrated for the V267A variant in Figure S5, or a flattening of the denaturation curve was observed as shown for the V264A variant (Figure S5). The flattening of the curve is interpreted as a restructuring of the amyloid aggregate during the long incubation time of one week, which let us concentrate on the use of the denaturation measurements with one day incubation for the quantitative analysis. Although in the case of V264A the extracted ΔG value did not alter between the two type of measurements (Figure S5), the change of the denaturation curve indicates that in GuHCl only a pseudo-equilibrium is obtained. Another point worth mentioning is that highly reproducible denaturation curves were obtained by incubating the protein in GuHCl solution without shaking, while in presence of shaking the reproducibility was only moderate (data not shown).

### Solid state NMR of amyloid fibrils of ^15^N,^13^C-labeled HET-s(218–289) variants

All the solid-state NMR experiments were conducted on a AVANCE II Bruker 600 MHz (14. 1 T) spectrometer using a Bruker 3.2 mm probe. DARR spectra with 20 ms mixing time were obtained from ^15^N,^13^C-labeled HET-s(218–289) variants [82]. All the spectra were processed using Topspin 2.0 (Bruker Biospin), using a shifted cosine squared window function as indicated in the Figure captions and so called zero-filling was done to the next power of two in both dimensions. Automated baseline correction was applied in the direct dimension. For spectral analysis the software package CcpNMR Analysis [83] was used and the chemical shift list was taken from [56].

### Sequence analysis

HET-S homologs were recovered from available genome databases at ncbi (http://blast.ncbi.nlm.nih.gov/Blast.cgi) and jgi (http://genome.jgi-psf.org/) using BLAST searches. Multiple alignments were performed with CLUSTALW2 at http://www.ebi.ac.uk/Tools/msa/clustalw2/. The consensus sequence of the R1 and R2 repeats were generated using MEME at meme.nbcr.net/, [53]. The MEME output gives a graphical representation of the consensus sequence as a weighted consensus in which the size of the letter designating a given amino acid is proportional to the conservation of the residue in the different sequences used to generate the motif. The size of the character reflects the information content measured in bits.

## Supporting Information

Figure S1 **Alignment of N-terminal region of HET-s/HET-S homologs.** The N-terminal regions (1–34/35) of the sequences listed in File S1 have been aligned with ClustalW2. The HET-s and HET-S sequences are boxed in red. At position 33 (H33 in HET-S and P33 in HET-s), no sequence is of the HET-s type and most sequences are of the HET-S type. In addition to H, amino acids found at that position are R, Q and N. It was shown that H33R, H33Q and H33N HET-S variants retain *het-S* specificity [51]; [52]. The sole exception is the Nh-mpIV-4 sequence from *Nectria haematococca* showing a L at that position. The H33L mutation in HET-S leads to the *het-s* specificity [51], [52]. In addition this sequence contains a deletion in the N-terminal region, making it possible that this sequence corresponds to a HET-s homolog.
(TIF)

Figure S2 **Experimental setting used to measure [Het-s]-propagation rate.** A row of 6 wild-type and mutant [Het-s*] subcultures are inoculated on solid medium and the first strains of the row is infected with wild-type [Het-s] (grey arrow) and confronted to HET-S tester strains, the prion propagates from one subculture to another. The barrage line in the confrontation zone with the HET-S tester shows the progression of the prion infection at the time of contact with the HET-S tester, that is about 10 hours after infection of the first strain in the row. The distance measured between the two white diamonds is the distance reported in Figure 3.
(TIF)

Figure S3 **2D Solid state NMR spectra of Ala variants of HET-s(218–289) amyloid fibrils for variants K229A to V267A.** The 2D DARR solid state NMR spectra of ^15^N,^13^C-labeled Ala variants of HET-S(218–289) amyloid fibrils are shown on top of the corresponding spectrum of wild-type HET-s(218–289) amyloid fibrils, the latter which contour lines are color-coded black. These are the same spectra as in Figure 4 with some cross peak assignment. The comparison between the 2D DARR of the Ala variant amyloid fibrils and the wild-type HET-s(218–289) amyloid fibrils indicates the preservation of the β-solenoid fold of all the Ala variants with small structural changes close to the replaced amino acid side chains as evidenced by small chemical shift perturbations upon Ala replacement. A significant chemical shift change is documented if the cross peak did shift approximately half a line width or more. In the following for each Ala variant these chemical shift perturbations are described: for K229A there is only a slight chemical shift change observed for resonances of V264. For I231A a substantial amount of spatial local chemical shift perturbations are observed including A228, K229, D230, T233, V239, L241, T266, V267, V268, V275, and I277 as well as the spatial not close residues E235, V244, A247, A248, K270, R274, and L276 did show also small chemical shift perturbations. For V239A only the spatially close residues I231, T233, R274, and I271 show chemical shift perturbations. For Q240A only R274, V239, V244, and I277 show slight chemical shift changes; for L241A: A228, K229, I231, E235, Q240, and I277, as well as the structurally further away positioned S273 and V264; for N262A: V264, I231, T261, S263, and I277, as well as the structurally further away positioned T266, and K270; for V264A: only A228 and I277; and for E265A nothing significantly. For the hydrophobic core residue V267A perturbed chemical shifts are of the core residues K229, I231, I277, V268, T233, and T266 and the spatially not close residue V244; For E272A no significant shifts are observed, for the Thr/Ser ladder residue S273A, chemical shift perturbations are observed for its partner T233, for R236, R274, and I277, respectively. For R274A as well as for N279A, no significant chemical shifts are observed. For the Ala replacement of the hydrophobic core residue I277 perturbed chemical shifts are of residues V264 and R274. For the single variants F286A, W287A, as well as D288A the shifts of the close in space residues V239 (only for F286A and W287A), Q240, V244, and R274 (and I277 for F286A) are altered.
(PDF)

Figure S4 **2D Solid state NMR spectra of Ala variants of HET-s(218–289) amyloid fibrils for variants E272A to D288A.** The 2D DARR solid state NMR spectra of ^15^N,^13^C-labeled Ala variants of HET-S(218–289) amyloid fibrils are shown on top of the corresponding spectrum of wild-type HET-s(218–289) amyloid fibrils, the latter which contour lines are color-coded black. These are the same spectra as in Figure 4 with some cross peak assignment.
(PDF)

Figure S5 **HET-s(218–289) V264A and V267A amyloid denaturation curves after 1 day and 1 week of incubation in GuHCl.** The GuHCl denaturation curve of amyloid fibrils of HET-s(218–289) V264A and V267A (as indicated) were measured by the OD_500_ (y-axis) at various GuHCl concentration (x-axis) after incubation in the corresponding GuHCl buffers for one day (black data, 1 d) or one week (green data, 1 w), respectively. While the extracted ΔG values are very similar for both types of measurement, in the case of V264A a flattening of the denaturation curve upon incubation in GuHCl for one week is evident indicating that during the long incubation restructuring of the amyloid may start to happen.
(TIF)

File S1 **List of HET-S homologs identified in different fungal species.**
(XLS)

## References

[1] Maury CP (2009) Self-propagating beta-sheet polypeptide structures as prebiotic informational molecular entities: the amyloid world. Orig Life Evol Biosph 39: 141–150. 19301141 10.1007/s11084-009-9165-6

[2] Greenwald J, Riek R (2010) Biology of amyloid: structure, function, and regulation. Structure 18: 1244–1260. 20947013 10.1016/j.str.2010.08.009

[3] Greenwald J, Riek R (2012) On the possible amyloid origin of protein folds. J Mol Biol 421: 417–426. 22542525 10.1016/j.jmb.2012.04.015

[4] Eichner T, Radford SE (2011) A diversity of assembly mechanisms of a generic amyloid fold. Mol Cell 43: 8–18. 21726806 10.1016/j.molcel.2011.05.012

[5] Monsellier E, Chiti F (2007) Prevention of amyloid-like aggregation as a driving force of protein evolution. EMBO Rep 8: 737–742. 17668004 10.1038/sj.embor.7401034 PMC1978086

[6] Blanco LP, Evans ML, Smith DR, Badtke MP, Chapman MR (2012) Diversity, biogenesis and function of microbial amyloids. Trends Microbiol 20: 66–73. 22197327 10.1016/j.tim.2011.11.005 PMC3278576

[7] Shewmaker F, McGlinchey RP, Wickner RB (2011) Structural insights into functional and pathological amyloid. J Biol Chem 286: 16533–16540. 21454545 10.1074/jbc.R111.227108 PMC3089495

[8] Chiti F, Dobson CM (2006) Protein misfolding, functional amyloid, and human disease. Annu Rev Biochem 75: 333–366. 16756495 10.1146/annurev.biochem.75.101304.123901

[9] Eisenberg D, Jucker M (2012) The amyloid state of proteins in human diseases. Cell 148: 1188–1203. 22424229 10.1016/j.cell.2012.02.022 PMC3353745

[10] Lansbury PT (1994) Mechanism of scrapie replication. Science 265: 1510. 10.1126/science.8079159 8079159

[11] Walker LC, LeVine H 3rd (2012) Corruption and spread of pathogenic proteins in neurodegenerative diseases. J Biol Chem 287: 33109–33115. 22879600 10.1074/jbc.R112.399378 PMC3460416

[12] Moreno-Gonzalez I, Soto C (2011) Misfolded protein aggregates: mechanisms, structures and potential for disease transmission. Semin Cell Dev Biol 22: 482–487. 21571086 10.1016/j.semcdb.2011.04.002 PMC3175247

[13] Toyama BH, Weissman JS (2011) Amyloid structure: conformational diversity and consequences. Annu Rev Biochem 80: 557–585. 21456964 10.1146/annurev-biochem-090908-120656 PMC3817101

[14] Colby DW, Prusiner SB (2011) Prions. Cold Spring Harb Perspect Biol 3: a006833. 21421910 10.1101/cshperspect.a006833 PMC3003464

[15] Liebman SW, Chernoff YO (2012) Prions in yeast. Genetics 191: 1014–1072. 10.1534/genetics.111.137760 PMC3415993 22879407

[16] Saupe SJ (2011) The [Het-s] prion of Podospora anserina and its role in heterokaryon incompatibility. Semin Cell Dev Biol 22: 460–468. 21334447 10.1016/j.semcdb.2011.02.019

[17] Coustou V, Deleu C, Saupe S, Begueret J (1997) The protein product of the het-s heterokaryon incompatibility gene of the fungus Podospora anserina behaves as a prion analog. Proc Natl Acad Sci U S A 94: 9773–9778. 9275200 10.1073/pnas.94.18.9773 PMC23266

[18] Debets AJ, Dalstra HJ, Slakhorst M, Koopmanschap B, Hoekstra RF, et al (2012) High natural prevalence of a fungal prion. Proc Natl Acad Sci U S A 109: 10432–10437. 22691498 10.1073/pnas.1205333109 PMC3387057

[19] Rizet G (1952) Les phénomènes de barrage chez Podospora anserina. I. Analyse de barrage entre les souches s et S. Rev Cytol Biol Veg 13: 51–92.

[20] Bidard F, Clave C, Saupe SJ (2013) The Transcriptional Response to Nonself in the Fungus Podospora anserina. G3 (Bethesda) 3: 1015–1030. 23589521 10.1534/g3.113.006262 PMC3689799

[21] Daskalov A, Paoletti M, Ness F, Saupe SJ (2012) Genomic Clustering and Homology between HET-S and the NWD2 STAND Protein in Various Fungal Genomes. Plos One 7: e34854. 22493719 10.1371/journal.pone.0034854 PMC3321046

[22] Paoletti M, Saupe SJ (2009) Fungal incompatibility: evolutionary origin in pathogen defense? Bioessays 31: 1201–1210. 19795412 10.1002/bies.200900085

[23] Beisson-Schecroun J (1962) Incompatibilité cellulaire et interactions nucléocytoplamsiques dans les phénomènes de barrage chez le Podospora anserina. Ann Genet 4: 3–50. 13970337

[24] Dalstra HJ, Swart K, Debets AJ, Saupe SJ, Hoekstra RF (2003) Sexual transmission of the [Het-S] prion leads to meiotic drive in Podospora anserina. Proc Natl Acad Sci U S A 100: 6616–6621. 12719532 10.1073/pnas.1030058100 PMC164496

[25] Balguerie A, Dos Reis S, Ritter C, Chaignepain S, Coulary-Salin B, et al (2003) Domain organization and structure-function relationship of the HET-s prion protein of Podospora anserina. Embo J 22: 2071–2081. 12727874 10.1093/emboj/cdg213 PMC156085

[26] Greenwald J, Buhtz C, Ritter C, Kwiatkowski W, Choe S, et al (2010) The mechanism of prion inhibition by HET-S. Mol Cell 38: 889–899. 20620958 10.1016/j.molcel.2010.05.019 PMC3507513

[27] Ritter C, Maddelein ML, Siemer AB, Luhrs T, Ernst M, et al (2005) Correlation of structural elements and infectivity of the HET-s prion. Nature 435: 844–848. 15944710 10.1038/nature03793 PMC1567094

[28] Van Melckebeke H, Wasmer C, Lange A, Ab E, Loquet A, et al (2010) Atomic-resolution three-dimensional structure of HET-s(218–289) amyloid fibrils by solid-state NMR spectroscopy. J Am Chem Soc 132: 13765–13775. 20828131 10.1021/ja104213j

[29] Wasmer C, Lange A, Van Melckebeke H, Siemer AB, Riek R, et al (2008) Amyloid fibrils of the HET-s(218–289) prion form a beta solenoid with a triangular hydrophobic core. Science 319: 1523–1526. 18339938 10.1126/science.1151839

[30] Seuring C, Greenwald J, Wasmer C, Wepf R, Saupe SJ, et al (2012) The mechanism of toxicity in HET-S/HET-s prion incompatibility. PLoS Biol 10: e1001451. 23300377 10.1371/journal.pbio.1001451 PMC3531502

[31] Saupe SJ, Daskalov A (2012) The [Het-s] Prion, an Amyloid Fold as a Cell Death Activation Trigger. PLoS Pathog 8: e1002687. 22654661 10.1371/journal.ppat.1002687 PMC3359984

[32] Mathur V, Seuring C, Riek R, Saupe SJ, Liebman SW (2012) Localization of HET-S to the cell periphery, not to [Het-s] aggregates, is associated with [Het-s]-HET-S toxicity. Mol Cell Biol 32: 139–153. 22037764 10.1128/MCB.06125-11 PMC3255711

[33] Cai X, Chen J, Xu H, Liu S, Jiang QX, et al (2014) Prion-like Polymerization Underlies Signal Transduction in Antiviral Immune Defense and Inflammasome Activation. Cell 156: 1207–1222. 24630723 10.1016/j.cell.2014.01.063 PMC4034535

[34] Mizuno N, Baxa U, Steven AC (2011) Structural dependence of HET-s amyloid fibril infectivity assessed by cryoelectron microscopy. Proc Natl Acad Sci U S A 108: 3252–3257. 21300906 10.1073/pnas.1011342108 PMC3044374

[35] Van der Nest MA, Olson A, Lind M, Velez H, Dalman K, et al (2014) Distribution and evolution of het gene homologs in the basidiomycota. Fungal Genet Biol 64: 45–57. 24380733 10.1016/j.fgb.2013.12.007

[36] Gendoo DM, Harrison PM (2011) Origins and evolution of the HET-s prion-forming protein: searching for other amyloid-forming solenoids. Plos One 6: e27342. 22096554 10.1371/journal.pone.0027342 PMC3214033

[37] Benkemoun L, Ness F, Sabate R, Ceschin J, Breton A, et al (2011) Two structurally similar fungal prions efficiently cross-seed in vivo but form distinct polymers when coexpressed. Mol Microbiol 82: 1392–1405. 22050595 10.1111/j.1365-2958.2011.07893.x

[38] Wasmer C, Zimmer A, Sabate R, Soragni A, Saupe SJ, et al (2010) Structural similarity between the prion domain of HET-s and a homologue can explain amyloid cross-seeding in spite of limited sequence identity. J Mol Biol 402: 311–325. 20600104 10.1016/j.jmb.2010.06.053

[39] Petkova AT, Leapman RD, Guo Z, Yau WM, Mattson MP, et al (2005) Self-propagating, molecular-level polymorphism in Alzheimer's beta-amyloid fibrils. Science 307: 262–265. 15653506 10.1126/science.1105850

[40] Wasmer C, Benkemoun L, Sabate R, Steinmetz MO, Coulary-Salin B, et al (2009) Solid-state NMR spectroscopy reveals that E. coli inclusion bodies of HET-s(218–289) are amyloids. Angew Chem Int Ed Engl 48: 4858–4860. 19472238 10.1002/anie.200806100

[41] Sabate R, Baxa U, Benkemoun L, Sanchez de Groot N, Coulary-Salin B, et al (2007) Prion and non-prion amyloids of the HET-s prion forming domain. J Mol Biol 370: 768–783. 17532341 10.1016/j.jmb.2007.05.014

[42] Wasmer C, Soragni A, Sabate R, Lange A, Riek R, et al (2008) Infectious and noninfectious amyloids of the HET-s(218–289) prion have different NMR spectra. Angew Chem Int Ed Engl 47: 5839–5841. 18548467 10.1002/anie.200704896

[43] Wan W, Bian W, McDonald M, Kijac A, Wemmer DE, et al (2013) Heterogeneous seeding of a prion structure by a generic amyloid form of the fungal prion-forming domain HET-s(218–289). J Biol Chem 288: 29604–29612. 23986444 10.1074/jbc.M113.505511 PMC3795258

[44] Wan W, Wille H, Stohr J, Baxa U, Prusiner SB, et al (2012) Degradation of fungal prion HET-s(218–289) induces formation of a generic amyloid fold. Biophys J 102: 2339–2344. 22677387 10.1016/j.bpj.2012.04.011 PMC3353098

[45] Ferguson N, Becker J, Tidow H, Tremmel S, Sharpe TD, et al (2006) General structural motifs of amyloid protofilaments. Proc Natl Acad Sci U S A 103: 16248–16253. 17060612 10.1073/pnas.0607815103 PMC1637568

[46] Williams AD, Shivaprasad S, Wetzel R (2006) Alanine scanning mutagenesis of Abeta(1–40) amyloid fibril stability. J Mol Biol 357: 1283–1294. 16476445 10.1016/j.jmb.2006.01.041

[47] Ross ED, Edskes HK, Terry MJ, Wickner RB (2005) Primary sequence independence for prion formation. Proc Natl Acad Sci U S A 102: 12825–12830. 16123127 10.1073/pnas.0506136102 PMC1200301

[48] Derkatch IL, Chernoff YO, Kushnirov VV, Inge-Vechtomov SG, Liebman SW (1996) Genesis and variability of [PSI] prion factors in Saccharomyces cerevisiae. Genetics 144: 1375–1386. 8978027 10.1093/genetics/144.4.1375 PMC1207691

[49] Tanaka M, Chien P, Naber N, Cooke R, Weissman JS (2004) Conformational variations in an infectious protein determine prion strain differences. Nature 428: 323–328. 15029196 10.1038/nature02392

[50] Marchante R, Rowe M, Zenthon J, Howard MJ, Tuite MF (2013) Structural Definition Is Important for the Propagation of the Yeast [PSI(+)] Prion. Mol Cell 50: 675–685. 23746351 10.1016/j.molcel.2013.05.010 PMC3679450

[51] Coustou V, Deleu C, Saupe SJ, Begueret J (1999) Mutational analysis of the [Het-s] prion analog of Podospora anserina. A short N-terminal peptide allows prion propagation. Genetics 153: 1629–1640. 10581272 10.1093/genetics/153.4.1629 PMC1460872

[52] Deleu C, Clave C, Begueret J (1993) A single amino acid difference is sufficient to elicit vegetative incompatibility in the fungus Podospora anserina. Genetics 135: 45–52. 8224826 10.1093/genetics/135.1.45 PMC1205625

[53] Bailey TL, Boden M, Buske FA, Frith M, Grant CE, et al (2009) MEME SUITE: tools for motif discovery and searching. Nucleic Acids Res 37: W202–208. 19458158 10.1093/nar/gkp335 PMC2703892

[54] Benkemoun L, Sabate R, Malato L, Dos Reis S, Dalstra H, et al (2006) Methods for the in vivo and in vitro analysis of [Het-s] prion infectivity. Methods 39: 61–67. 16750391 10.1016/j.ymeth.2006.04.006

[55] Malato L, Dos Reis S, Benkemoun L, Sabate R, Saupe SJ (2007) Role of Hsp104 in the propagation and inheritance of the [Het-s] prion. Mol Biol Cell 18: 4803–4812. 17881723 10.1091/mbc.E07-07-0657 PMC2096600

[56] Siemer AB, Ritter C, Ernst M, Riek R, Meier BH (2005) High-resolution solid-state NMR spectroscopy of the prion protein HET-s in its amyloid conformation. Angew Chem Int Ed Engl 44: 2441–2444. 15770629 10.1002/anie.200462952

[57] Creighton TE (1993) Proteins: Structures and Molecular Properties, 2nd edition. New York: Freedman.

[58] Luisi DL, Snow CD, Lin JJ, Hendsch ZS, Tidor B, et al (2003) Surface salt bridges, double-mutant cycles, and protein stability: an experimental and computational analysis of the interaction of the Asp 23 side chain with the N-terminus of the N-terminal domain of the ribosomal protein l9. Biochemistry 42: 7050–7060. 12795600 10.1021/bi027202n

[59] Marqusee S, Sauer RT (1994) Contributions of a hydrogen bond/salt bridge network to the stability of secondary and tertiary structure in lambda repressor. Protein Sci 3: 2217–2225. 7756981 10.1002/pro.5560031207 PMC2142769

[60] Schreiber G, Fersht AR (1995) Energetics of protein-protein interactions: analysis of the barnase-barstar interface by single mutations and double mutant cycles. J Mol Biol 248: 478–486. 7739054 10.1016/s0022-2836(95)80064-6

[61] Santoro MM, Bolen DW (1988) Unfolding free energy changes determined by the linear extrapolation method. 1. Unfolding of phenylmethanesulfonyl alpha-chymotrypsin using different denaturants. Biochemistry 27: 8063–8068. 3233195 10.1021/bi00421a014

[62] Wang WaR, C.J., editor (2010) Aggregation of therapeutic proteins. Hoboken, New Jersey: Wiley.

[63] Whitten ST, Wooll JO, Razeghifard R, Garcia-Moreno EB, Hilser VJ (2001) The origin of pH-dependent changes in m-values for the denaturant-induced unfolding of proteins. J Mol Biol 309: 1165–1175. 11399086 10.1006/jmbi.2001.4726

[64] Batey S, Clarke J (2006) Apparent cooperativity in the folding of multidomain proteins depends on the relative rates of folding of the constituent domains. Proc Natl Acad Sci U S A 103: 18113–18118. 17108086 10.1073/pnas.0604580103 PMC1636339

[65] Myers JK, Pace CN, Scholtz JM (1995) Denaturant m values and heat capacity changes: relation to changes in accessible surface areas of protein unfolding. Protein Sci 4: 2138–2148. 8535251 10.1002/pro.5560041020 PMC2142997

[66] Kajava AV, Baxa U, Steven AC (2010) Beta arcades: recurring motifs in naturally occurring and disease-related amyloid fibrils. FASEB J 24: 1311–1319. 20032312 10.1096/fj.09-145979 PMC2879952

[67] Tanaka M, Collins SR, Toyama BH, Weissman JS (2006) The physical basis of how prion conformations determine strain phenotypes. Nature 442: 585–589. 16810177 10.1038/nature04922

[68] Legname G, Nguyen HO, Peretz D, Cohen FE, DeArmond SJ, et al (2006) Continuum of prion protein structures enciphers a multitude of prion isolate-specified phenotypes. Proc Natl Acad Sci U S A 103: 19105–19110. 17142317 10.1073/pnas.0608970103 PMC1748184

[69] Colby DW, Giles K, Legname G, Wille H, Baskakov IV, et al (2009) Design and construction of diverse mammalian prion strains. Proc Natl Acad Sci U S A 106: 20417–20422. 19915150 10.1073/pnas.0910350106 PMC2787151

[70] Friedman R, Caflisch A (2013) Wild type and mutants of the HET-s(218–289) prion show different flexibility at fibrillar ends: A simulation study. Proteins 82: 399–404. 24038616 10.1002/prot.24402

[71] Chiti F, Webster P, Taddei N, Clark A, Stefani M, et al (1999) Designing conditions for in vitro formation of amyloid protofilaments and fibrils. Proc Natl Acad Sci U S A 96: 3590–3594. 10097081 10.1073/pnas.96.7.3590 PMC22338

[72] Nelson R, Eisenberg D (2006) Recent atomic models of amyloid fibril structure. Curr Opin Struct Biol 16: 260–265. 16563741 10.1016/j.sbi.2006.03.007

[73] Nelson R, Sawaya MR, Balbirnie M, Madsen AO, Riekel C, et al (2005) Structure of the cross-beta spine of amyloid-like fibrils. Nature 435: 773–778. 15944695 10.1038/nature03680 PMC1479801

[74] Sawaya MR, Sambashivan S, Nelson R, Ivanova MI, Sievers SA, et al (2007) Atomic structures of amyloid cross-beta spines reveal varied steric zippers. Nature 447: 453–457. 17468747 10.1038/nature05695

[75] Margittai M, Langen R (2006) Side chain-dependent stacking modulates tau filament structure. J Biol Chem 281: 37820–37827. 17023423 10.1074/jbc.M605336200

[76] Tjernberg L, Hosia W, Bark N, Thyberg J, Johansson J (2002) Charge attraction and beta propensity are necessary for amyloid fibril formation from tetrapeptides. J Biol Chem 277: 43243–43246. 12215440 10.1074/jbc.M205570200

[77] Zanuy D, Nussinov R (2003) The sequence dependence of fiber organization. A comparative molecular dynamics study of the islet amyloid polypeptide segments 22–27 and 22–29. J Mol Biol 329: 565–584. 12767835 10.1016/s0022-2836(03)00491-1

[78] Fernandez-Escamilla AM, Rousseau F, Schymkowitz J, Serrano L (2004) Prediction of sequence-dependent and mutational effects on the aggregation of peptides and proteins. Nat Biotechnol 22: 1302–1306. 15361882 10.1038/nbt1012

[79] Tartaglia GG, Pawar AP, Campioni S, Dobson CM, Chiti F, et al (2008) Prediction of aggregation-prone regions in structured proteins. J Mol Biol 380: 425–436. 18514226 10.1016/j.jmb.2008.05.013

[80] Trovato A, Chiti F, Maritan A, Seno F (2006) Insight into the structure of amyloid fibrils from the analysis of globular proteins. PLoS Comput Biol 2: e170. 17173479 10.1371/journal.pcbi.0020170 PMC1698942

[81] El-Khoury R, Sellem CH, Coppin E, Boivin A, Maas MF, et al (2008) Gene deletion and allelic replacement in the filamentous fungus Podospora anserina. Curr Genet 53: 249–258. 18265986 10.1007/s00294-008-0180-3

[82] Takegoshi K, Nakamura S, Terao T (2001) 13C-1H dipolar-assisted rotational resonance in magic-angle spinning NMR. Phys Lett 344: 631–637.

[83] Vranken WF, Boucher W, Stevens TJ, Fogh RH, Pajon A, et al (2005) The CCPN data model for NMR spectroscopy: development of a software pipeline. Proteins 59: 687–696. 15815974 10.1002/prot.20449

