# Supplementary figures and images for: Contribution of Specific Residues of the β-Solenoid Fold to HET-s Prion Function, Amyloid Structure and Stability
Source: PLoS Pathog. 2014 Jun 12;10(6):e1004158. doi: 10.1371/journal.ppat.1004158 (PMC4055769; doi:10.1371/journal.ppat.1004158)

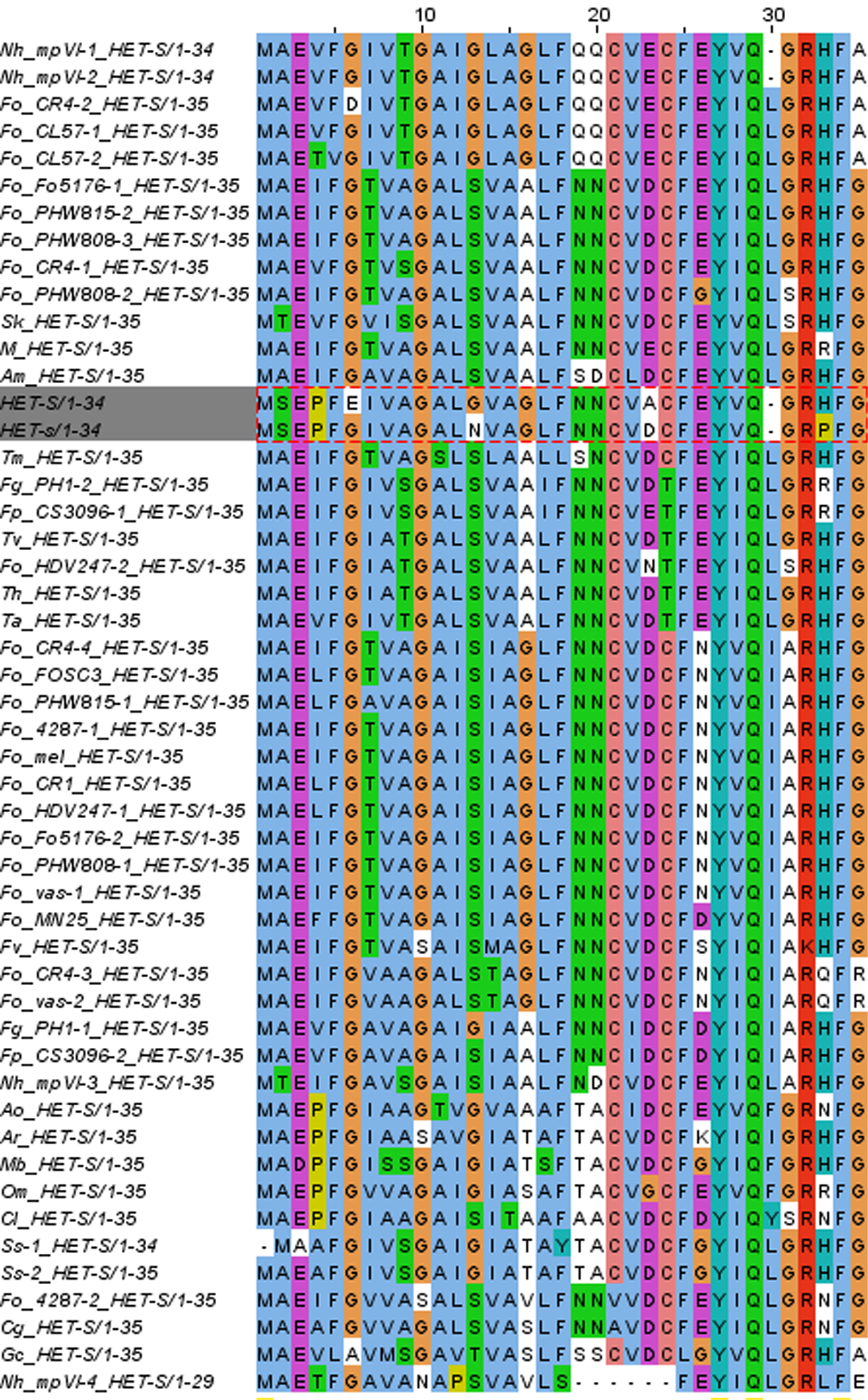

Supplement: Figure S1 — Alignment of N-terminal region of HET-s/HET-S homologs. The N-terminal regions (1–34/35) of the sequences listed in File S1 have been aligned with ClustalW2. The HET-s and HET-S sequences are boxed in red. At position 33 (H33 in HET-S and P33 in HET-s), no sequence is of the HET-s type and most sequences are of the HET-S type. In addition to H, amino acids found at that position are R, Q and N. It was shown that H33R, H33Q and H33N HET-S variants retain het-S specificity [51]; [52]. The sole exception is the Nh-mpIV-4 sequence from Nectria haematococca showing a L at that position. The H33L mutation in HET-S leads to the het-s specificity [51], [52]. In addition this sequence contains a deletion in the N-terminal region, making it possible that this sequence corresponds to a HET-s homolog. (TIF) [file ppat.1004158.s001.tif]

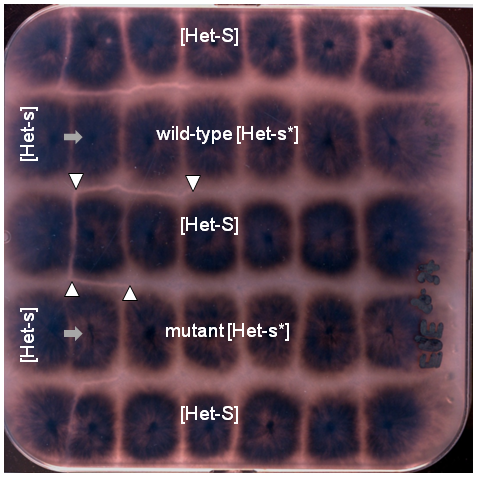

Supplement: Figure S2 — Experimental setting used to measure [Het-s]-propagation rate. A row of 6 wild-type and mutant [Het-s*] subcultures are inoculated on solid medium and the first strains of the row is infected with wild-type [Het-s] (grey arrow) and confronted to HET-S tester strains, the prion propagates from one subculture to another. The barrage line in the confrontation zone with the HET-S tester shows the progression of the prion infection at the time of contact with the HET-S tester, that is about 10 hours after infection of the first strain in the row. The distance measured between the two white diamonds is the distance reported in Figure 3. (TIF) [file ppat.1004158.s002.tif]

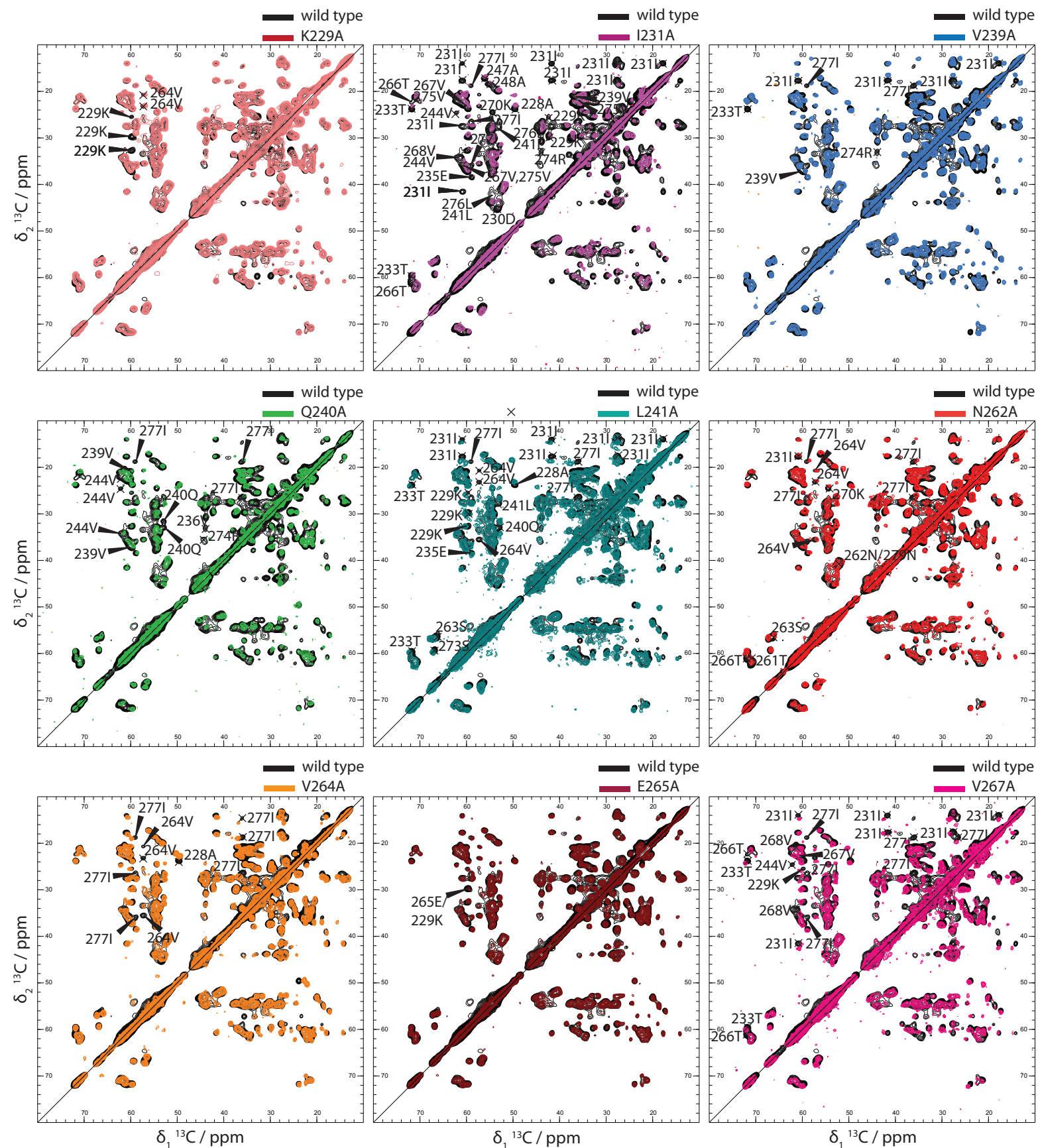

Supplement: Figure S3 — 2D Solid state NMR spectra of Ala variants of HET-s(218–289) amyloid fibrils for variants K229A to V267A. The 2D DARR solid state NMR spectra of 15N,13C-labeled Ala variants of HET-S(218–289) amyloid fibrils are shown on top of the corresponding spectrum of wild-type HET-s(218–289) amyloid fibrils, the latter which contour lines are color-coded black. These are the same spectra as in Figure 4 with some cross peak assignment. The comparison between the 2D DARR of the Ala variant amyloid fibrils and the wild-type HET-s(218–289) amyloid fibrils indicates the preservation of the β-solenoid fold of all the Ala variants with small structural changes close to the replaced amino acid side chains as evidenced by small chemical shift perturbations upon Ala replacement. A significant chemical shift change is documented if the cross peak did shift approximately half a line width or more. In the following for each Ala variant these chemical shift perturbations are described: for K229A there is only a slight chemical shift change observed for resonances of V264. For I231A a substantial amount of spatial local chemical shift perturbations are observed including A228, K229, D230, T233, V239, L241, T266, V267, V268, V275, and I277 as well as the spatial not close residues E235, V244, A247, A248, K270, R274, and L276 did show also small chemical shift perturbations. For V239A only the spatially close residues I231, T233, R274, and I271 show chemical shift perturbations. For Q240A only R274, V239, V244, and I277 show slight chemical shift changes; for L241A: A228, K229, I231, E235, Q240, and I277, as well as the structurally further away positioned S273 and V264; for N262A: V264, I231, T261, S263, and I277, as well as the structurally further away positioned T266, and K270; for V264A: only A228 and I277; and for E265A nothing significantly. For the hydrophobic core residue V267A perturbed chemical shifts are of the core residues K229, I231, I277, V268, T233, and T266 and the spatially [file ppat.1004158.s003.pdf]

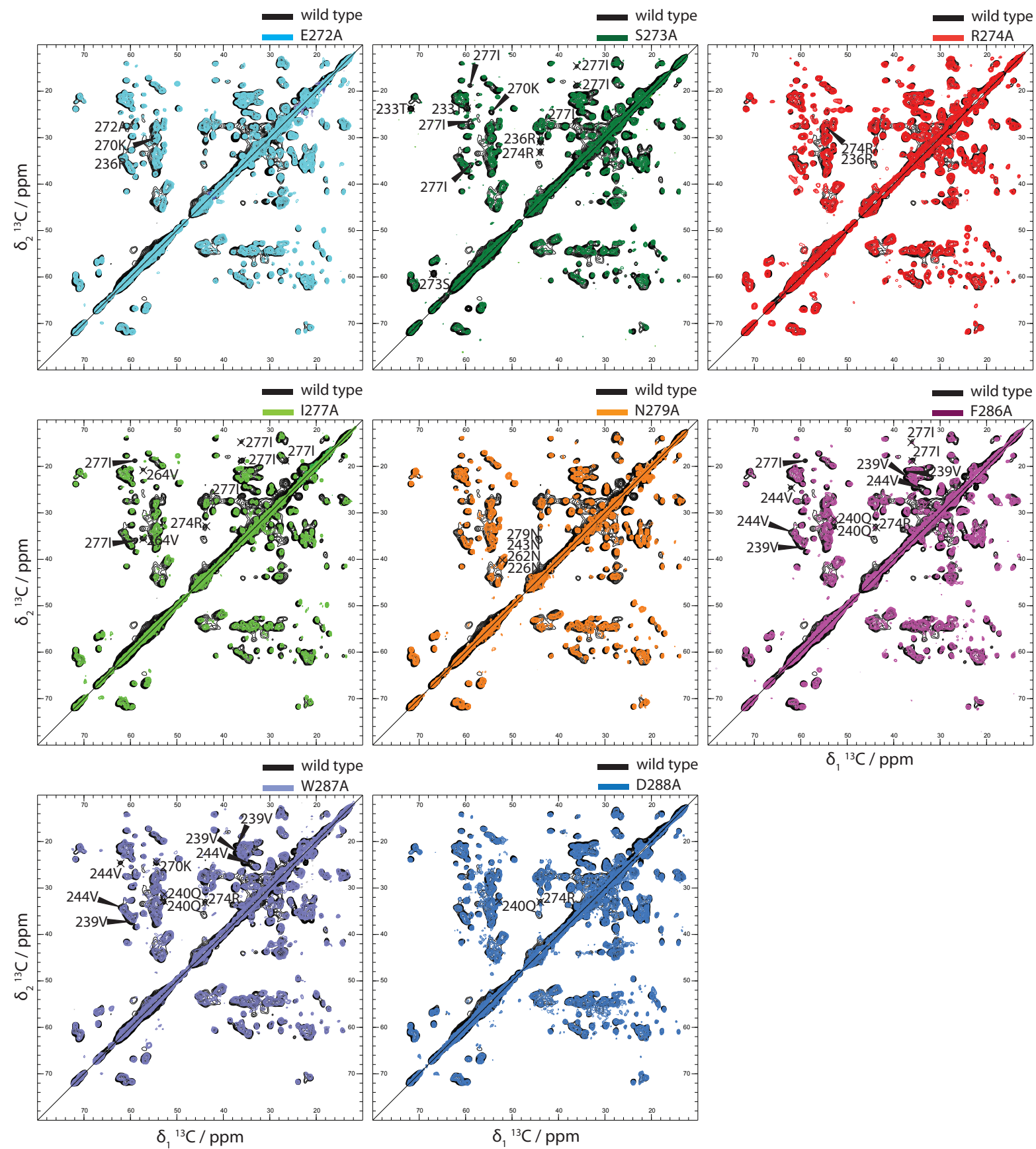

Supplement: Figure S4 — 2D Solid state NMR spectra of Ala variants of HET-s(218–289) amyloid fibrils for variants E272A to D288A. The 2D DARR solid state NMR spectra of 15N,13C-labeled Ala variants of HET-S(218–289) amyloid fibrils are shown on top of the corresponding spectrum of wild-type HET-s(218–289) amyloid fibrils, the latter which contour lines are color-coded black. These are the same spectra as in Figure 4 with some cross peak assignment. (PDF) [file ppat.1004158.s004.pdf]

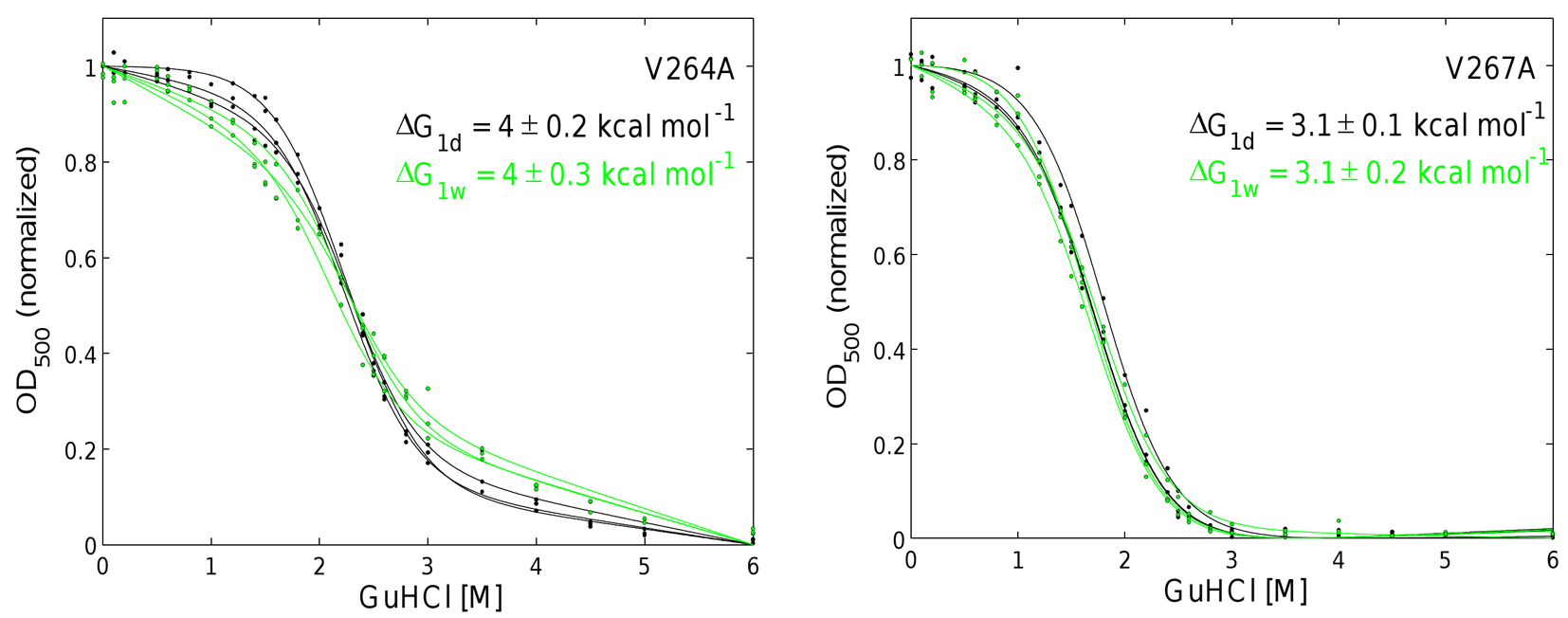

Supplement: Figure S5 — HET-s(218–289) V264A and V267A amyloid denaturation curves after 1 day and 1 week of incubation in GuHCl. The GuHCl denaturation curve of amyloid fibrils of HET-s(218–289) V264A and V267A (as indicated) were measured by the OD500 (y-axis) at various GuHCl concentration (x-axis) after incubation in the corresponding GuHCl buffers for one day (black data, 1 d) or one week (green data, 1 w), respectively. While the extracted ΔG values are very similar for both types of measurement, in the case of V264A a flattening of the denaturation curve upon incubation in GuHCl for one week is evident indicating that during the long incubation restructuring of the amyloid may start to happen. (TIF) [file ppat.1004158.s005.tif]
